# Visualizing modules of coordinated structural brain atrophy during the course of conversion to Alzheimer's disease by applying methodology from gene co-expression analysis

**DOI:** 10.1016/j.nicl.2019.101957

**Published:** 2019-07-25

**Authors:** Kenichiro Sato, Tatsuo Mano, Hiroshi Matsuda, Michio Senda, Ryoko Ihara, Kazushi Suzuki, Hiroyuki Arai, Kenji Ishii, Kengo Ito, Takeshi Ikeuchi, Ryozo Kuwano, Tatsushi Toda, Takeshi Iwatsubo, Atsushi Iwata, Japanese Alzheimer's Disease Neuroimaging Initiative

**Affiliations:** a Department of Neurology, Graduate School of Medicine, University of Tokyo, Japan; b National Center for Neurology and Psychiatry, Kodaira, Japan; c Kobe City Medical Center General Hospital, Kobe, Japan; d Department of Geriatrics & Gerontology, Division of Brain Science, Institute of Development, Aging and Cancer (IDAC), Tohoku University, Japan; e Tokyo Metropolitan Institute of Gerontology, Tokyo, Japan; f National Center for Geriatrics and Gerontology, Obu, Japan; g Niigata University, Niigata, Japan; h Unit for Early and Exploratory Clinical Development, The University of Tokyo Hospital, Tokyo, Japan; i Department of Neuropathology, Graduate School of Medicine, University of Tokyo, Japan

**Keywords:** Brain atrophy, Mild cognitive impairment, Alzheimer's disease module, Hierarchical clustering, Connectivity

## Abstract

**Objective:**

We aimed to identify modularized structural atrophy of brain regions with a high degree of connectivity and its longitudinal changes associated with the progression of Alzheimer's disease (AD) using weighted gene co-expression network analysis (WGCNA), which is an unsupervised hierarchical clustering method originally used in genetic analysis.

**Methods:**

We included participants with late mild cognitive impairment (MCI) at baseline from the Japanese Alzheimer's Disease Neuroimaging Initiative (J-ADNI) study. We imputed normalized and *Z*-transformed structural volume or cortical thickness data of 164 parcellated brain regions/structures based on the calculations of the *FreeSurfer* software. We applied the WGCNA to extract modules with highly interconnected structural atrophic patterns and examined the correlation between the identified modules and clinical AD progression.

**Results:**

We included 204 participants from the baseline dataset, and performed a follow-up with 100 in the 36-month dataset of MCI cohort participants from the J-ADNI. In the univariate correlation or variable importance analysis, baseline atrophy in temporal lobe regions/structures significantly predicted clinical AD progression. In the WGCNA consensus analysis, co-atrophy modules associated with MCI conversion were first distributed in the temporal lobe and subsequently extended to adjacent parietal cortical regions in the following 36 months.

**Conclusions:**

We identified coordinated modules of brain atrophy and demonstrated their longitudinal extension along with the clinical course of AD progression using WGCNA, which showed a good correspondence with previous pathological studies of the tau propagation theory. Our results suggest the potential applicability of this methodology, originating from genetic analyses, for the surrogate visualization of the underlying pathological progression in neurodegenerative diseases not limited to AD.

## Highlights

• Applying origin-of-genetic unsupervised clustering to structural MRI from J-ADNI.
• We identified highly-interconnected modules associated with AD progression.
• Extension of co-atrophy modules corresponded with tau propagation theory in AD.
• This method has potential for visualizing underlying pathological progression.
• Application to other neurodegenerative diseases not limited to AD is expected.

## 1 Introduction

Regional brain atrophy indicates a decline in its corresponding function; therefore, the structural features of brain atrophy associated with the disease course are important hallmarks to accurately predict the conversion of mild cognitive impairment (MCI) to Alzheimer's disease (AD) and the subsequent disease progression. To illustrate, regional atrophy in the temporal lobe predicts MCI conversion to AD (Tapiola et al., 2008; Risacher et al., 2009; Querbes et al., 2009; Misra et al., 2009; Ferreira et al., 2011; Falahati et al., 2017), indicating the underlying AD pathology (Braak and Braak, 1991; Frisoni et al., 2010; Jucker and Walker, 2011).

Magnetic resonance imaging (MRI) studies on structural differences between MCI converters and non-converters have typically been based on regional measurements, in which two or more groups in each region/structure, such as in voxel-based morphometry (Ashburner and Friston, 2000), were compared independently using a *t*-test or generalized linear model. However, since brain regions are interconnected and neurodegeneration in some neurodegenerative diseases continuously propagates through an anatomical network within the brain in a “prion-like” manner (Frost and Diamond, 2010; Brundin et al., 2010), such an inter-regional interaction-independent approach may potentially overlook coordinated changes underlying anatomically- or functionally-interconnected regions/structures.

The connectome approach, a method to overcome interaction-independent approaches, has been well investigated to facilitate the understanding of interconnected changes between brain regions (Hagmann et al., 2008; Bullmore and Sporns, 2009; Stam, 2014). With regard to AD/MCI, structural and functional network analysis has revealed the reduced connectivity metrics between temporal, parietal, and frontal lobes in AD (Yao et al., 2010; Griffa et al., 2013; Zhu et al., 2014; Prescott et al., 2016; Filippi et al., 2018). Although these studies report connectivity metrics between individual nodes, they have not fully considered modularization of multiple inter-correlated nodes. Assuming that the intercorrelation-based modules can be modeled as bundles of nodes influenced or changed to a similar degree with each other, longitudinal changes in the distribution of such “coordinated” modules, demonstrating clinical AD progression associated regional/structural changes (including atrophy), might indicate the involvement of underlying pathology in a significantly interconnected manner. In other words, by modularizing highly interconnected regions/structures and measuring their association with the longitudinal clinical prognosis, it may be possible to visualize the underlying pathological propagation of AD indirectly.

Therefore, we employed a weighted gene co-expression network analysis (WGCNA), which is an unsupervised hierarchical clustering method originating from genetic analysis (Zhang and Horvath, 2005; Langfelder and Horvath, 2007; Langfelder and Horvath, 2008; Langfelder and Horvath, 2014). Originally, the WGCNA was established in genome-wide gene expression studies, which enables us to identify relevant “gene modules”, i.e., highly interconnected genes that may be incorporated into underlying biological pathways. This method can also be applied to brain imaging data, since it has an identical data structure as that of the gene expression dataset, having a large number of predefined and normalizable features with relatively limited sample sizes. This method has previously been applied to functional MRI (fMRI) data (Mumford et al., 2010), in an attempt to introduce it to the field of neuroradiology for the first time. It had subsequently reported reliable detection of more number of parcellated and spatially-focused modules than via independent component analysis, and provided reasonably adequate results in the identification of for inter-regional connections of the brain. We aimed to identify intra-modular brain regions/structures with significantly similar structural (including atrophic) changes across the samples, agnostic of anatomical/functional knowledge, by applying this method to the structural brain MRI data of MCI participants from the Japanese Alzheimer's Disease Neuroimaging Initiative (J-ADNI) (Iwatsubo et al., 2018; Iwata et al., 2018), which is a multi-center prospective observational study for the progression of MCI and mild AD in the Japanese population. Furthermore, we evaluated correlations between the modules and clinical prognostic metrics associated with MCI conversion and ADAS-cog13 progression, over a longitudinal time-course. To the best of our knowledge, this is the first-ever attempt to use WGCNA to assess structural changes in the AD brain.

## 2 Methods

### 2.1 Sample datasets

We used the J-ADNI dataset downloaded from the National Bioscience Database Center (NBDC) with the approval of its data access committee (https://humandbs.biosciencedbc.jp/en/hum0043-v1). General inclusion criteria for MCI participants in J-ADNI are as follows: participants themselves or participants' family have complained of memory disturbances, their age ranges from 60 to 84 years at baseline; they are native Japanese speakers; their total Mini Mental State Examination (MMSE) scores fall in the range of 24–30, and their Clinical Dementia Rating (CDR) score and memory box of CDR should be 0.5 and 0.5 or greater, respectively. The follow-up period for the J-ADNI dataset was 3 years (36 months) for NC and MCI, and 2 years (24 months) for AD participants. Participants having MCI at baseline were referred to as MCI cohort participants, and those who were cognitively normal (CN) at baseline as the CN cohort participants. We included the baseline dataset and follow-up at 36 months dataset for all MCI or CN cohort participants whose preprocessed MRI data were available.

### 2.2 Clinical variables

We selected the Alzheimer's Disease Assessment Scale – cognitive subscale 13 (ADAS-cog13) and MMSE, from the several neuropsychological tests used in the J-ADNI cohort, to assess the longitudinal cognitive and functional status of participants. Since not all participant did not undergo an ADAS test at 36 months (their last timing of ADAS-cog13: median 36 months (IQR: 24–36)), we used the ADAS progression speed instead of raw differences in ADAS-cog13 scores. ADAS-cog13 progression speed score was calculated as follows: progression speed = (score at the last visit for ADAS-cog13 (up to 36 months) – baseline score)/(number of months between baseline and the last visit for ADAS). Additionally, we obtained the number of MCI cohort participants who experienced conversion to mild AD during the observational period, which was judged by the study investigators in each facility: the subjects with mild AD had to successfully satisfy the National Institute of Neurological and Communicative Disorders and Stroke–Alzheimer's Disease and Related Disorders Association criteria for probable AD (McKhann et al., 1984). Further detailed features of MCI participants who converted to AD or those who did not are reported in our previous report (Sato et al., 2019). Other clinical and laboratory data used for analysis included their sex, age, serum creatinine level, creatinine clearance calculated from the Cockroft–Gault equation (Cockcroft and Gault, 1976), cerebrospinal fluid (CSF) amyloid-beta 1–42 (Abeta42) level, CSF phosphorylated tau (p-tau) level, presence of *APOE* ε4 alleles, and amyloid-PET positivity.

### 2.3 Structural brain MRI data processing

**Fig. 1:**
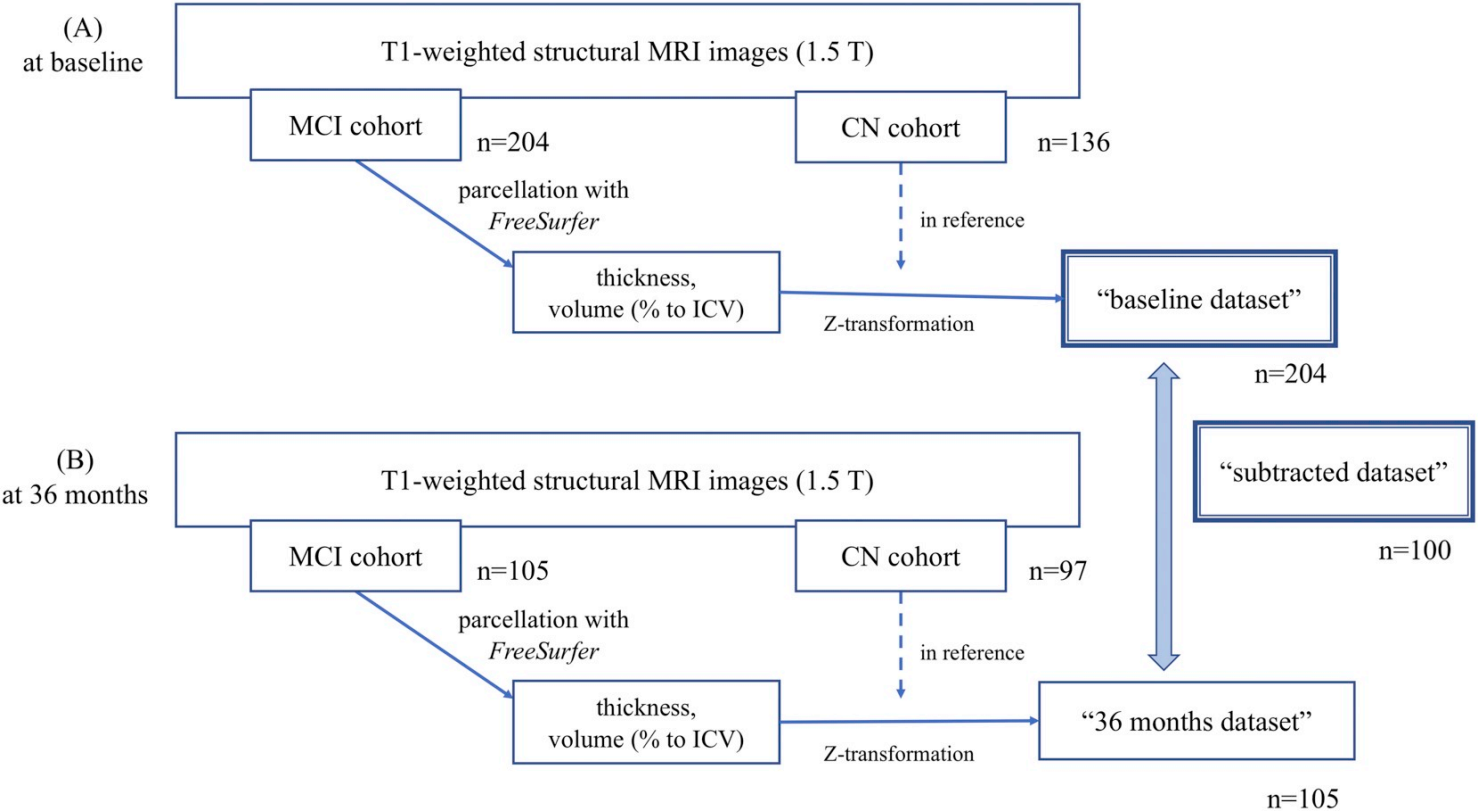
Data processing workflow. Regarding Z-transformation of both the baseline and 36-month datasets, we included those who were MCI at baseline (A; *n* = 204 in baseline dataset and *n* = 105 in 36-months dataset), and used those who were CN at baseline (B; *n* = 136 in baseline dataset and *n* = 97 in 36-months dataset) as a reference. In cases whose MRI data are available in both the baseline and 36 months datasets, the subtracted Z-score is derived (*n* = 100) to observe longitudinal changes in atrophy (here we refer to this as “36-months dataset subtracted from screening dataset results”).

We used T1-weighted MRI (1.5-T) image data obtained during screening (baseline dataset) and at the 36-month follow-up (36-months dataset), both of which had been preprocessed using *FreeSurfer* software (Version 5.1) (Fischl, 2012) and uploaded to the NBDC database (filenames: “lh.aparc.stats.tsv”, “rh.aparc.stats.tsv”, “aseg.stats.tsv”, and “wmparc.stats.tsv”). We obtained 164 regional/structural measured values in addition to the total intracranial volume (ICV), the mean cortical thickness value (mm) of the right and left sides of 34 cortical regions defined by the Desikan–Killiany atlas (Desikan et al., 2006), right and left subcortical volume (mm^3^) of these 34 parcellated regions, and the volume (mm^3^) of another 28 subcortical brain structures listed in Table 1. We excluded cerebellar structural volumes from the analysis because AD pathology should not cause cerebellar atrophy. Data processing (Fig. 1) was carried out by first normalizing the values of the structural volume by representing it as a percentage of ICV in each case (Voevodskaya et al., 2014); however, mean cortical thickness was not normalized (Westman et al., 2013). Since the distribution of the thickness/volume values would not always be normal and to avoid the influence of potential outliers, we transformed the unnormalized mean cortical thickness and regional/structural volume (normalized to ICV) to a robust Z score in reference to the data of the cognitive normal control (CN) cohort participants. The Z score was calculated in each region/structure in the MRI data of each MCI participant in the baseline dataset (here we refer to the derived dataset as the “baseline dataset” as shown in Fig. 1) via the following equation: robust Z = [raw value – (median value of reference (CN))] × 1.3489/[interquartile range (IQR) of reference (CN)], where 1.3489 is derived from the normal probability distribution from 25% to 75% to normalize interquartile range (IQR). We used this robust Z for normalization as opposed to the conventional Z normalization technique, since the volume/thickness distribution did not particularly obey normal distribution, even though the distribution of the data was not significantly skewed. Additionally, we tried to avoid the influence of potential outliers, which may occur partly due to the current study criteria of including every participant's MRI data regardless of their actual degree of atrophy, by using robust Z. The same procedure was used for the MRI dataset obtained at the 36-month follow-up (referred to as the “36-months dataset”). Since we tried to examine the net progression during the 3 years, there was a non-negligible percentage (16–18%) of amyloid positive subjects among CN subgroup at baseline, we used data from CN at 36 months as the reference for that from MCI at 36 months. As summarized in Fig. 1, to carry out *Z*-conversion for both of the baseline dataset and 36-months dataset, we included those who were MCI at baseline (*n* = 204 in the baseline dataset and *n* = 105 in the 36-months dataset), and using participants who were CN at baseline (*n* = 136 in the baseline dataset and *n* = 97 in the 36-months dataset) as a reference. In 100 cases where MRI data were available both in the baseline dataset and 36-months dataset, we derived a subtracted Z score to analyze longitudinal changes in atrophy (we refer to this derived dataset as the “subtracted dataset”). The Z scores in the baseline dataset represent the relative atrophic measurements of MCI brains at baseline compared to CN brains, and Z scores in the subtracted dataset represent the longitudinal changes of relative atrophic metrics of MCI brains during the 36-month follow-up.

**Table 1:** List of regions/structures and their abbreviations.

Thirty-four regions (Desikan–Killiany atlas): gray matter/white matter	Laterality	Abbreviation	Other subcortical structures	Laterality	Abbreviation
Bank of superior temporal sulcus	R/L	Bankssts	Lateral ventricle	R/L	Lateral.Ventricle
Caudal anterior cingulate cortex	R/L	Caudalanteriorcingulate	Thalamus proper	R/L	Thalamus.Proper
Caudal middle frontal gyrus	R/L	Caudalmiddlefrontal	Caudate	R/L	Caudate
Cuneus cortex	R/L	Cuneus	Putamen	R/L	Putamen
Entorhinal cortex	R/L	Entorhinal	Pallidum	R/L	Pallidum
Fusiform gyrus	R/L	Fusiform	Third ventricle	–	3rd.Ventricle
Inferior parietal cortex	R/L	Inferiorparietal	Hippocampus	R/L	Hippocampus
Inferior temporal gyrus	R/L	Inferiortemporal	Amygdala	R/L	Amygdala
Isthmus cingulate cortex	R/L	Isthmuscingulate	Accumbens area	R/L	Accumbens.area
Lateral occipital cortex	R/L	Lateraloccipital	Ventral diencephalon	R/L	VentralDC
Lateral orbitofrontal cortex	R/L	Lateralorbitofrontal	White matter hypointensities	–	WM.hypointensities
Lingual gyrus	R/L	Lingual	Non-white matter hypointensities	–	non.WM.hypointensities
Medial orbitofrontal cortex	R/L	Medialorbitofrontal	Corpus callosum: anterior	–	CC_Anterior
Middle temporal gyrus	R/L	Middletemporal	Corpus callosum: mid-anterior	–	CC_Mid_Anterior
Parahippocampal gyrus	R/L	Parahippocampal	Corpus callosum: central	–	CC_Central
Paracentral lobule	R/L	Paracentral	Corpus callosum: mid-posterior	–	CC_Mid_Posterior
Pars opercularis	R/L	Parsopercularis	Corpus callosum: posterior	–	CC_Posterior
Pars orbitalis	R/L	Parsorbitalis	Unsegmented white matter	R/L	UnsegmentedWhiteMatter
Pars triangularis	R/L	Parstriangularis			
Pericalcarine cortex	R/L	Pericalcarine			
Postcentral gyrus	R/L	Postcentral			
Posterior cingulate cortex	R/L	Posteriorcingulate			
Precentral gyrus	R/L	Precentral			
Precuneus cortex	R/L	Precuneus			
Rostral anterior cingulate cortex	R/L	Rostralanteriorcingulate			
Rostral middle frontal gyrus	R/L	Rostralmiddlefrontal			
Superior frontal gyrus	R/L	Superiorfrontal			
Superior parietal gyrus	R/L	Superiorparietal			
Superior temporal gyrus	R/L	Superiortemporal			
Supramarginal gyrus	R/L	Supramarginal			
Frontal pole	R/L	Frontalpole			
Temporal pole	R/L	Temporalpole			
Transverse temporal cortex	R/L	Transversetemporal			
Insular	R/L	Insula			

The 164 regions/structures comprise 34 right and left gray and white matter regions, two unsegmented white matter regions, and 26 subcortical structures/parcellations.

Suffix of “.x” or “.y” denotes the laterality of right or left, respectively. For example, “entorhinal.x” represent the right-sided gray matter of the entorhinal cortex. A prefix of “Right.” or “Left.” refers to the laterality of regions/structures. The prefix of “wm.rh.” or “wm.lh.” means “right-side white matter” or “left-side white matter” respectively.

### 2.4 Statistical analyses

All statistical analyses were performed using R version 3.3.3 (https://www.r-project.org) and its packages. We used the Fisher exact test to test categorical data and Wilcoxon rank sum test for continuous data, unless mentioned otherwise. Differences with a *p*-value of <.05 were considered statistically significant. To perform multiple testing correction, we used the Benjamini & Hochberg (BH) method (Storey and Tibshirani, 2003). In the univariate correlation analysis, Spearman's rank correlation test was used to evaluate correlations between each of the normalized regional cortical thickness values or regional/structural volumes and clinical features. The correlation coefficient (rho) is represented as a heatmap.

Furthermore, we evaluated each region of importance according to the importance of variables associated with the clinical deterioration endpoint (MCI conversion and progression in ADAS-cog13 speed) using the R package, “*caret*” (Kuhn, 2008). We used a support vector machine algorithm to discriminate conversion or to regress for ADAS progression speed using the Z scores for each region/structure (Klöppel et al., 2008). Importance variables for conversion was obtained as the normalized [0−100] area under curve of discriminating ROC for each region/structure, while the same for ADAS progression speed was obtained as the R^2^ in the ADAS progression speed of observed versus predicted, from each region/structure. Tenfold cross validation and hyperparameter tuning was performed with the *caret* package function.

### 2.5 WGCNA analysis

The WGCNA analysis was performed using the R package, “*WGCNA*” (version 1.61) (Langfelder and Horvath, 2008). Originally, WGCNA was formulated to identify gene modules, groups of highly interconnected genes that may be incorporated into biological pathways. First, the baseline and subtracted dataset as preprocessed above were imputed for pairwise Spearman's rank correlation, between regional/structural atrophic Z scores, to construct signed networks. The adjacency matrix was defined using the ‘soft’-thresholding to weigh by a [0–1] number instead of 0/1, to emphasize large adjacencies while de-emphasizing smaller adjacencies. The power used in the soft thresholding is determined according to the scale-free topology criterion: the lowest power at which saturation is reached as long as it is above 0.80 in both datasets (Supplemental Fig. 2A) (Mumford et al., 2010). These networks were then tuned into Topological Overlap Matrices (TOM), and two TOMS were scaled across two datasets.

Since the purpose of this study was to compare the baseline J-ADNI dataset and its longitudinal changes, we performed a “consensus module” analysis (Langfelder and Horvath, 2007) to identify modules of which atrophic patterns within modules are preserved between two or more different networks, thereby enabling us to compare network results from different datasets (such as the baseline and subtracted datasets). Thus, the consensus TOM with preserved features between the two TOMs was calculated. The modularization of regions/structures was obtained by the hierarchical clustering of this consensus TOM, at which the minimal module size was set to two regions/structures to identify coordinated atrophy in as much detail as possible. TOM-based dissimilarity measure was then used to build network dendrograms, in which the modules represented the branches (Mumford et al., 2010). The cut-off threshold to merge close dendrogram branches, for the derived dendrogram, was set to a value of 0.2 or less, corresponding to 0.80 or more of similarity between modules; therefore, yielding the final modules (Supplemental Fig. 2B).

A module color was automatically allocated and the regions/structures not classified into any module were then bundled as the “gray module”. We analyzed the principle component within each module, and the first principle component, named as ‘module eigengene’ in the original article (Langfelder and Horvath, 2007), was used as a representative value to characterize each module. We then evaluated the correlation (Spearman's rank correlation) between the first principle component of the module and the baseline and prognostic clinical features. We used the R package, “*ggseg*” for visualization of the module results (Athanasia, 2019).

### 2.6 Code and data availability

As described above, the original data we used for this article can be downloaded from the National Bioscience Database Center (NBDC) (https://humandbs.biosciencedbc.jp/en/hum0043-v1).

### 2.7 Ethics

The study protocol was approved by the University of Tokyo ethics committee (11628).

## 3 Results

### 3.1 Correlation between clinical features and atrophic measurements in each region/structure

We included 204 MCI participants at baseline, of which, 100 participants were also included in the 3-year follow-up data from the J-ADNI study (Fig. 1). The basic clinical features of the 204 MCI cohort participants in comparison with the CN cohort participants (as a reference of atrophy) are summarized in Table 2. As previously reported (Iwatsubo et al., 2018), the MCI cohort participants were significantly older, received fewer years of education, had worse baseline cognitive scale scores, higher rates of *APOE* ε4 alleles, and higher amyloid-biomarker positivity (amyloid-PET and/or CSF Aβ_42_). Among them, 108/204 (52.94%) participants converted to AD during the 36-month follow-up period.

**Table 2:** Clinical features of CN and MCI participants at baseline.

	CN (*n* = 136) median/frequency	CN IQR/%	MCI (*n* = 204) median/frequency	MCI IQR/%	*p*
Baseline age (y/o)	67	(64–71)	74	(69–77.25)	<.001
Sex: Female	72/136	52.94%	103/204	50.49%	.740
Education (years)	14	(12–16)	12	(12–16)	.008
Baseline ADAS-cog13	7.5	(4.7–10)	20	(15.3–24.7)	<.001
Baseline MMSE	30	(29–30)	26	(25–28)	<.001
*APOE*ε4 allele(s)	1/32/103		16/89/97		<.001
Amyloid PET positivity	9/48	18.75%	35/55	63.64%	<.001
CSF Aβ (pg/mL)	467.9	(347.7–571.8)	297.5	(248.7–409.2)	<.001
CSF Aβ <333 pg/mL	8/50	16.00%	48/76	63.16%	<.001
CSF p-tau (pg/mL)	34.85	(32.27–40.21)	58.98	(39.75–78.72)	<.001
Baseline CCR (mL/min)	96.3	(78.37–114.0)	79.15	(66.1–94.04)	<.001
Conversion of MCI to AD during 3-year follow-up	–	–	108/204	52.94%	–
ADAS progression speed (delta scores/followed months)	−0.0194	(−0.0833–0.03818)	0.1833	(0.02502–0.3611)	<.001

There is a significant difference in most baseline clinical features between MCI and CN cohort participants, including baseline age. Since the CN data was used only for the purpose of providing a normal reference in *Z*-transformation, the differences in baseline age between MCI and CN cohort participants is permissible.

**Fig. 2:**
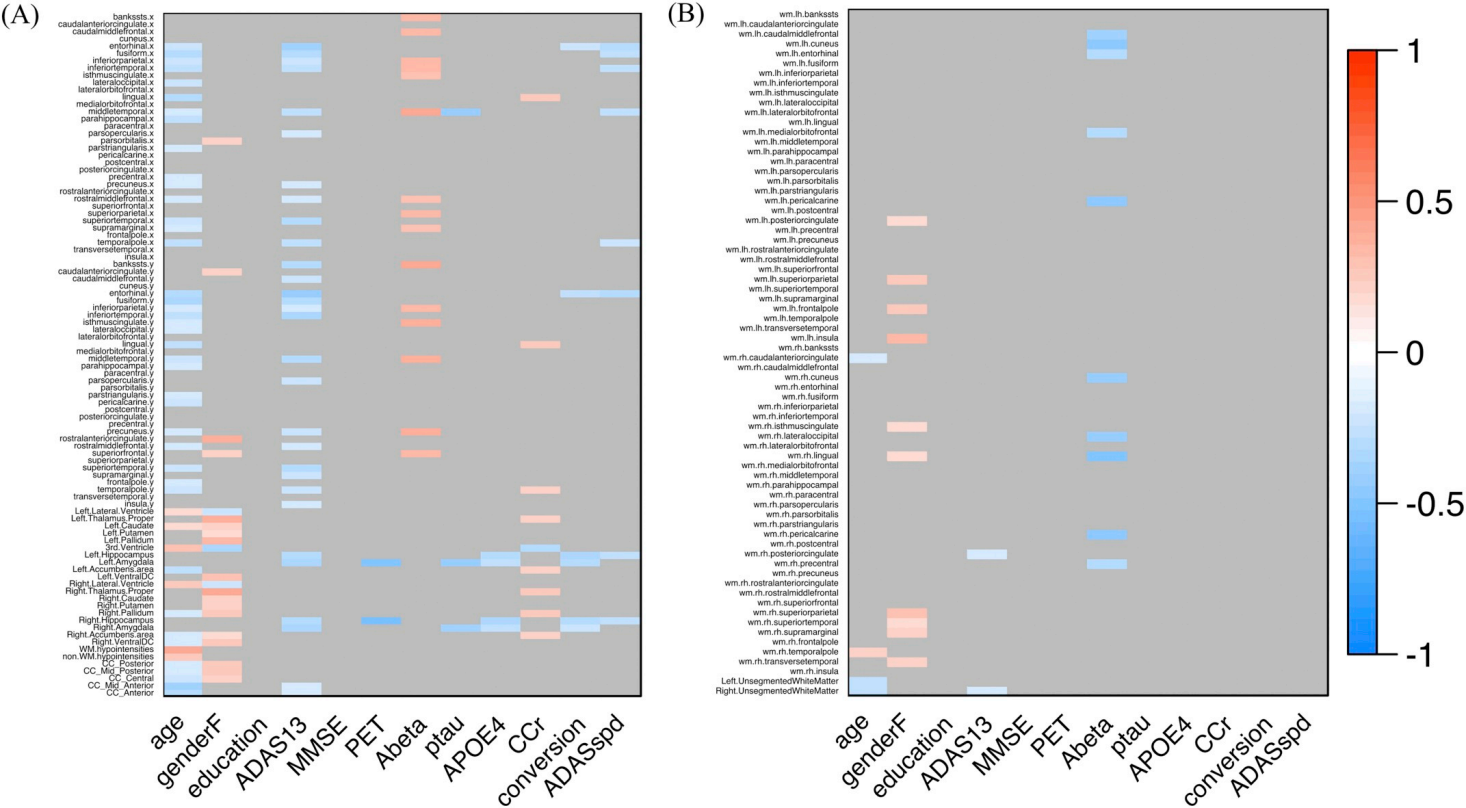
Correlation heatmap between regional/structural atrophy and clinical features (from the baseline dataset: *n* = 204). Heatmaps of the results of correlation analysis between the mean regional cortical thickness value (mm) or structural volume (percentage of the ICV) and clinical features in the baseline dataset. The heatmap color corresponds to a rho value (Spearman's rank correlation), from blue (corresponding to −1) to red (corresponding to +1). The raw significance is adjusted using the BH method (FDR), and non-significant items (*FDR >* *0.05*) are colored gray. The Y-axis on (A) denotes 34 bilateral cortical regions and other structures, and the Y-axis on (B) denotes the bilateral subcortical white matter of the 34 regions.

**Fig. 3:**
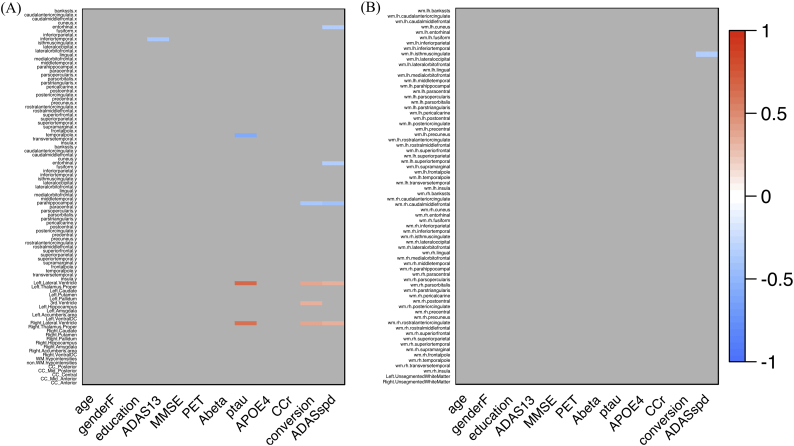
Correlation heatmap between regional/structural atrophy and clinical features (from the subtracted dataset: *n* = 100). Heatmaps of correlation analysis results between the mean regional cortical thickness value (mm) or structural volume (percentage of the ICV) and clinical features in the baseline dataset. The heatmap color corresponds to the rho value (Spearman's rank correlation), from blue (which corresponds to −1) to red (which corresponds to +1). The raw significance is adjusted using the BH method (FDR), and non-significant items (*FDR > 0.05*) are colored gray. The Y-axis on (A) denotes 34 bilateral cortical regions and other structures, and the Y-axis on (B) denotes the subcortical white matter of the 34 bilateral regions. (For interpretation of the references to color in this figure legend, the reader is referred to the web version of this article.)

To evaluate the basic relationship between regional/structural atrophy and AD progression, we analyzed the correlation between brain regional measurements (normalized) and clinical features, such as the incidence of conversion or ADAS-cog13 progression speed (Fig. 2, Fig. 3). Briefly, baseline regional/structural atrophy of the temporal and parietal lobe (Fig. 2) demonstrated significant correlation with the participants' baseline age, cognitive function, amyloid biomarkers (CSF Aβ_42_ and amyloid PET), *APOE* ε4 allele, and poorer future clinical deterioration (positive incidence of conversion or higher ADAS-cog13 progression speed within the 36 months) (Supplemental Table 1). Meanwhile, in the subtracted dataset, derived by subtracting Z scores of the 36-months dataset from that of the baseline (Fig. 3), the relative thickness reduction in the entorhinal cortex and para-hippocampal gyrus, and enlargement in the lateral and third ventricle were significantly associated with poorer clinical deterioration, for up to 36 months (Supplemental Table 1). These results demonstrate that features of structural abnormality on MRI in the MCI participants of the J-ADNI cohort are consistent with earlier MCI studies from other cohorts, showing either significant entorhinal and hippocampal (Tapiola et al., 2008) and medial temporal atrophy (Risacher et al., 2009; Ferreira et al., 2011) in MCI converters compared to non-converters, or significant difference in the volume of the left temporal lobe (Yang et al., 2012), hippocampal, parietal lobe, and ventricular volume (Sørensen et al., 2016) between MCI and AD.

**Supplemental Fig. 1:**
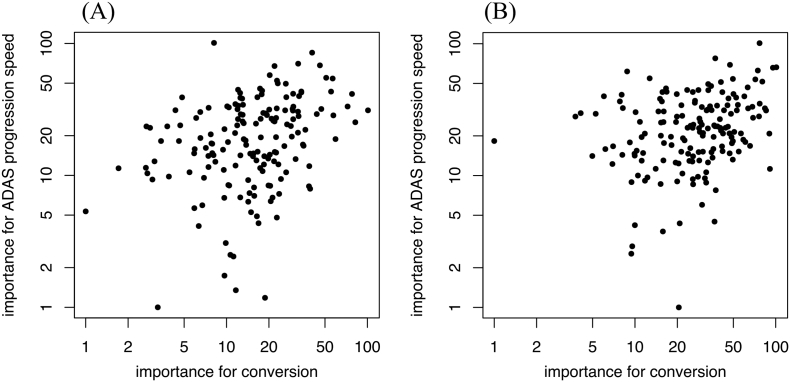
Scatter plot of variable importance for MCI conversion or ADAS progression speed. Importance +1 values are plotted on a logarithmic axis. (A) is a Y—Y plot of the baseline dataset (*n* = 204) between the importance for conversion and the importance for ADAS progression speed: rho = 0.339 (*p* < .001, Spearman's rank correlation). (B) is a Y—Y plot of the subtracted dataset (*n* = 100) between the importance for conversion and importance for ADAS progression speed: rho = 0.289 (*p* < .001). The variable importance shows an approximate positive correlation with each other.

We then performed multivariate analysis for each regional/structural association with clinical prognostic metrics. We avoided using linear regression, due to the prominent multicollinearity between right and left individual regions/structures or between adjacent regions/structures. Instead, we performed variable importance analysis by applying a support vector machine algorithm as observed in previous studies (Klöppel et al., 2008) and calculated the importance of variable in each region/structure in the regression for the clinical prognostic metrics (i.e., the incidence of MCI conversion and ADAS-cog13 progression speed) on both the baseline and subtracted datasets. In the baseline dataset, majority of the 20 highest regional/structural variable importance comprised of temporal lobe regions/structures (Supplemental Table 2), while other regions/structures, such as lateral ventricles or regions from parietal lobe rank, are listed as 20 highest values in the subtracted dataset (Supplemental Table 3). These results were consistent with the univariate correlation result as in Fig. 2, Fig. 3. Additionally, to confirm the clinical significance of variable importance, we analyzed the relationship between variable importance, and MCI conversion and ADAS-cog13 progression in the baseline dataset (Supplemental Fig. 1A) and on the subtracted dataset (Supplemental Fig. 1B), respectively. Across 164 regions/structures, there was a mild and significant correlation between each feature's importance for conversion and the importance for ADAS-cog13 progression speed: rho = 0.339 (*p* < .001, Spearman's rank correlation) in the baseline dataset and rho = 0.289 (*p* < .001) in the subtracted dataset, respectively.

### 3.2 Interconnectivity analysis using WGCNA

**Supplemental Fig. 2:**
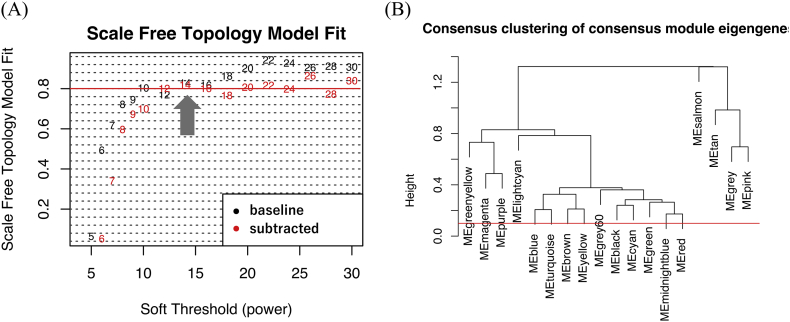
Scale-free topology criterion and Cluster dendrogram of the modules. (A) Schematic representation of the scale-free topology criterion. The soft-power threshold was determined as the lowest power at which saturation is reached as long as it is above 0.80 (shown with lateral red line) in both datasets (shown with a filled arrow). Consensus Topological Overlap Matrices (TOM) was then calculated from the adjacency matrices of both datasets. After conducting hierarchical clustering for the consensus TOM, close dendrogram branches were merged at a certain cut-off level, yielding the final module (B).

Since the univariate region-to-region analysis assumes that the changes in each region/structure are independent and does not allow coordinated changes of interconnected regions/structures that might indicate involvement of underlying pathological. We then applied the WGCNA to identify potentially interconnected regional/structural modules in a longitudinal time course. We first performed a “consensus module” analysis of the baseline and subtracted datasets (Langfelder and Horvath, 2007) to identify the modules commonly preserved in both. The networks from both datasets were constructed with a soft threshold power of 14, determined according to the scale-free topology criterion (Supplemental Fig. 2A). We then identified the modules in which atrophic patterns were preserved between these two networks. With regard to the module dendrogram derived from the consensus analysis, the cut-off threshold to merge adjacent dendrogram branches was adjusted from 0.10 to 0.20, yielding the same final modules (Supplemental Fig. 2B).

As a result, 46 (28.05%) regions/structures out of 164 regions/structures were allocated into 17 modules with a minimal module size of two regions/structures (Table 3). Unclassified regions were bundled together as a “gray” module. Cortical regions tended to be modularized within the same lobe (“brown” and “yellow” for temporal cortical regions; and “cyan”, “red”, and “midnightblue” for parietal cortical regions), and subcortical structures tended to be modularized bilaterally (“salmon” for bilateral lateral ventricles; “greenyellow” for bilateral thalamus; and “magenta”, “purple”, and “lightcyan” for bilateral basal ganglia).

**Table 3:**
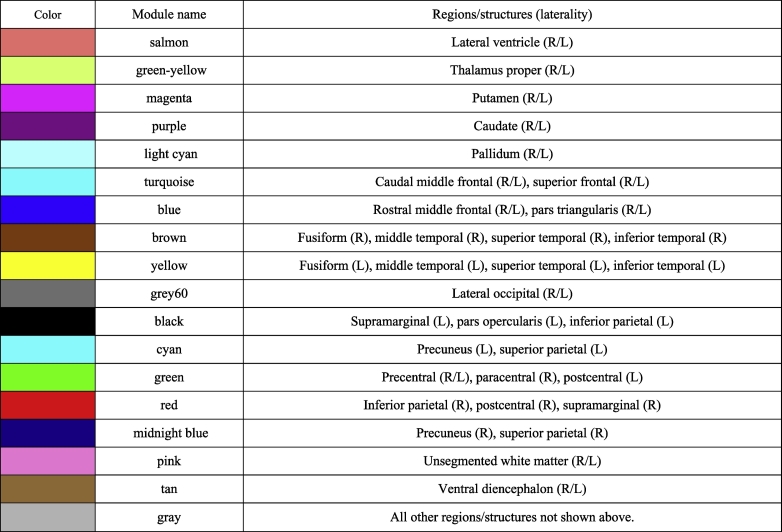
Regions/structures assigned to each module.

The “gray module” is an expedient label for unclassified regions/structures.

**Fig. 4:**
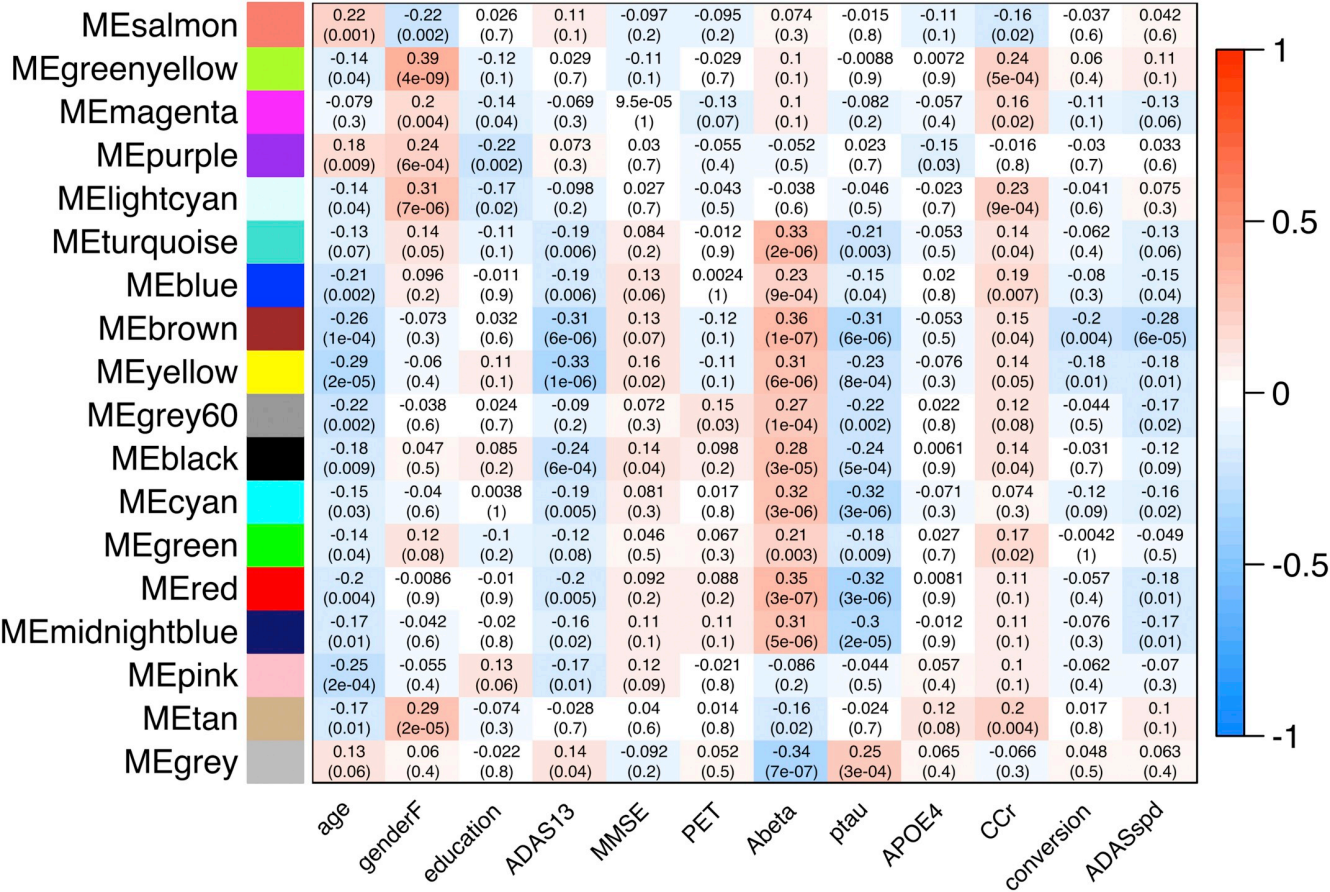
Correlation heatmap between clinical features and the modules' first principle component (from the baseline dataset: *n* = 204). Results of consensus analysis between the baseline and subtracted dataset, deriving preserved modules from the same. The heatmap shows the correlation between the first principle component of regional/structural measurements within each module (rows) from the baseline dataset and clinical features (columns). The heatmap color corresponds to the rho value (−1 to +1). The upper row in each table square denotes the rho value of the Spearman's rank correlation and the lower row denotes its *p*-value.

**Fig. 5:**
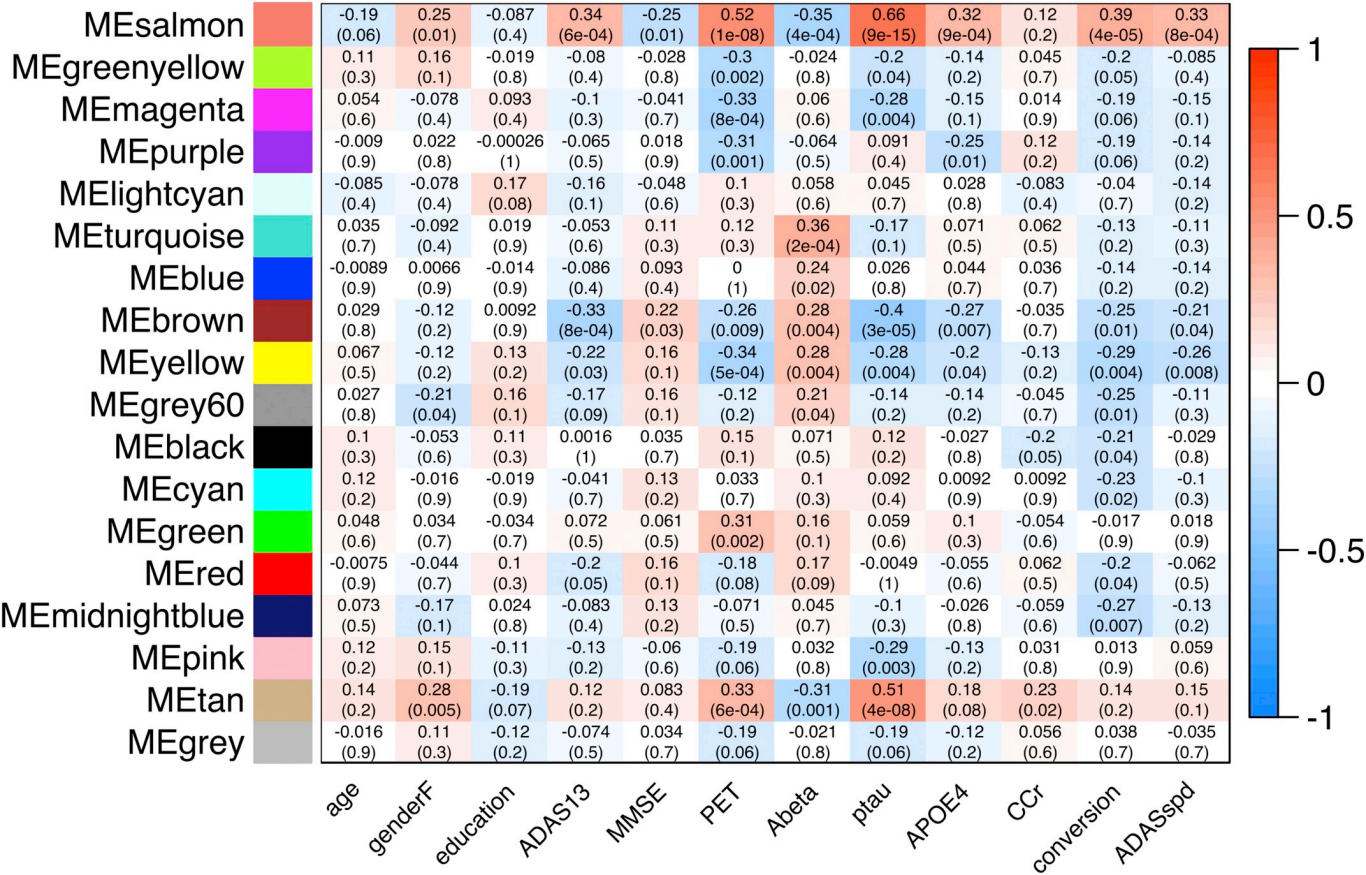
Correlation heatmap between clinical features and the modules' first principle component (from the subtracted dataset: *n* = 100). Results of consensus analysis between the baseline and subtracted dataset, deriving preserved modules from the same. The heatmap shows the correlation between the modules' first principle component of regional/structural measurements within each module (rows) from the subtracted dataset and clinical features. The heatmap color corresponds to the rho value (−1 to +1). The upper row in each table square denotes the rho value of the Spearman's rank correlation and the lower row denotes its *p*-value.

**Fig. 6:**
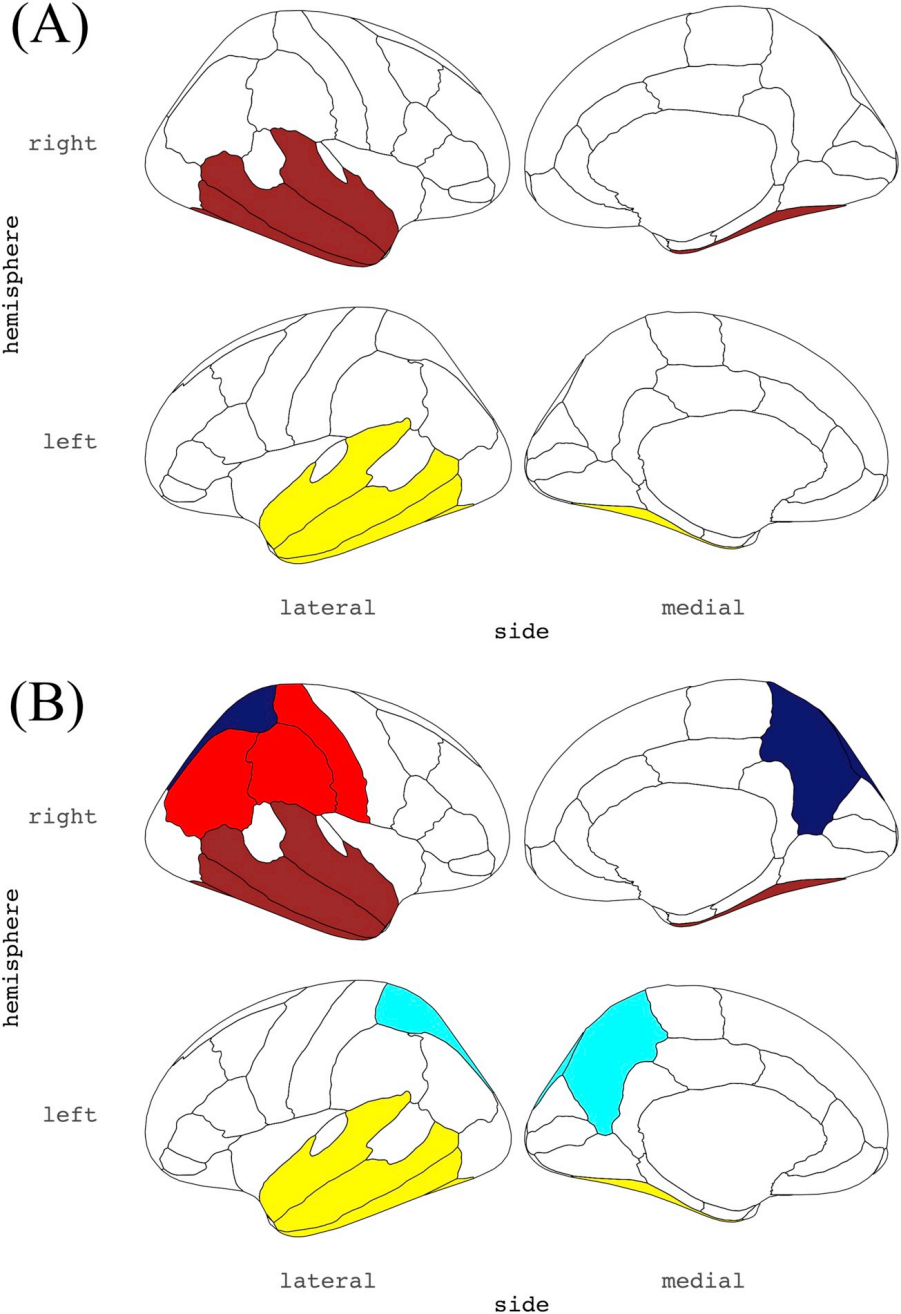
Distribution of the derived modules associated with the incidence of MCI conversion. (A) and (B) show the module distribution from the baseline and subtracted dataset, respectively; these modules are correlated with a higher incidence rate of MCI conversion during the 36 months of follow-up. Only cortical modules are shown. In each image (A & B), the left column shows the lateral cortex, and the right column shows the medial cortex. The co-atrophied modules at baseline (A) were distributed in bilateral temporal lobe (“yellow” and “brown”), implying that these regions showed similar levels of atrophy among all the parcellated regions/structures across samples. The distribution of modules showing similar level of atrophic change during the 36 months (B) then extended to the bilateral parietal lobe (“cyan”, “red”, and “midnightblue”). (For interpretation of the references to color in this figure legend, the reader is referred to the web version of this article.)

To identify modules associated with clinical metrics including MCI conversion and ADAS-cog13 progression, we derived each module's first principle component, followed by calculating its correlation with clinical features. The correlations are represented in the heatmap in Fig. 4 (for the baseline dataset) and Fig. 5 (for the subtracted dataset). Regarding the results in the baseline dataset (Fig. 4), atrophy in the regions of the temporal cortex (“brown” for right-sided and “yellow” for left-sided) demonstrated significantly correlated with poorer future clinical deterioration (MCI conversion and the ADAS-cog13 progression speed) (Fig. 6A). Modules such as the parietal (“cyan”, “red”, and “midnight blue”) and occipital cortex (“grey60”) were only associated with poorer ADAS-cog13 progression speed.

Furthermore, in the correlation of the first principle component and clinical features from the subtracted dataset (Fig. 5), changes in the degree of atrophy of the temporal cortex (“brown” and “yellow” module) or enlargement of the lateral ventricles (“salmon” module) during the 36 months were also significantly correlated with poorer clinical deterioration (Fig. 6B). Additionally, atrophic changes, primarily in the parietal and occipital cortices (“grey60”, “black”, “cyan”, “red”, and “midnight blue” modules) during the 36-month follow-up were also significantly correlated with the incidence of the conversion, while ADAS-cog13 progression speed was not correlated with these cortical modules in the subtracted dataset. Summarily, with regard to the longitudinal changes from baseline to 36 months, distribution of coordinated and grouped co-atrophic regions associated with conversion, first localized to the temporal cortex. However, over the period of 36 months it was observed in both temporal and parietal cortex.

## 4 Discussion

During the course of AD, along with underlying AD pathological progression (Braak and Braak, 1991; Frisoni et al., 2010; Jucker and Walker, 2011), abnormalities in amyloid biomarkers (Shaw et al., 2009) precedes structural changes in the brain changes (Tapiola et al., 2008; Querbes et al., 2009; Schuff et al., 2009), which extends from the medial temporal lobe to the parietal and frontal lobes, followed by a deterioration in cognitive function (Frisoni et al., 2010). Since the degree of atrophy directly correlates with the degree of pathological influence, including a reduction in the number of synapses and neuronal death, co-atrophied regions/structures (or regions/structures showing similar degrees of atrophy) should demonstrate approximately similar degrees of pathological influence. Our results show that coordinated atrophy at baseline was observed in the module of the temporal cortex, but not in that of the parietal cortex was associated with MCI conversion. However, coordinated net progression of atrophy in each of these modules, during the 36 months were associated with the MCI conversion, suggesting that the modular regions of coordinated atrophy associated with MCI conversion extended from the temporal to the parietal cortex. Based on the fact that pathological changes propagate through neuronal networks in several neurodegenerative diseases (Brundin et al., 2010), the extended distribution of such coordinated atrophy might correspond to the underlying AD pathological progression.

The WGCNA method has hardly ever been applied in the field of neuroradiological studies, except in an earlier weighted voxel coactivation network analysis (WVCNA) study (Mumford et al., 2010), which applied the same network construction and module identification methods to fMRI data. Our study is different from the earlier study in that we applied the methodological framework to structural brain MRI data, thereby demonstrating its potential applicability in inferring underlying pathological extension in the brain of neurodegenerative disease patients. The rationale in applying this methodology, originally used in genetic analysis, to neuroradiological data is because of the similarity in data structure as well as due to the problems that might be shared by researchers in the fields of neuroradiology and genetics: a large number of features with relatively limited sample size, coordinated, but not independent, changes within each feature, and the efficacy of using data-driven analysis. In a dataset with a large number of radiological features (such as the 1000 regions-of-interest within parcellated brain regions/structures in earlier literature (Hagmann et al., 2008)), comparison between two or more phenotypes (e.g., CN vs MCI vs AD), region-to-region univariate testing (regardless of whether it is a *t*-test, Wilcoxon rank sum test, analysis of variance, generalized linear regression, or some other method) is most frequently used, in which inter-regional interactive differences are not considered. Similarly, researchers in the field of genetics who investigate genome-wide studies (e.g., methylation microarrays, ChIP-seq, RNA-seq, proteomics, etc.) compare each gene or genomic feature between two or more of phenotypes (e.g., disease versus control) independently, making it difficult to recognize underlying interconnecting genetic changes along with hidden or unhidden biological pathways. WGCNA is a hierarchical clustering methodology that has been developed to address this problem (Langfelder and Horvath, 2007) by extracting highly interconnected gene groups and thereby elucidating the underlying biological pathways associated with the disease. If there were some latent interconnected structures mediated by underlying pathological involvement, within the brains of individuals with neurodegenerative diseases, it is expected that we can visualize them as co-atrophic module distributions using WGCNA. The modules associated with clinical deterioration of MCI (i.e., “brown”, “yellow”, “grey60”, “black”, “cyan”, “red”, and “midnight blue”) demonstrated atrophy in the temporal, parietal, and some parts of occipital and frontal cortex. These results are consistent with the well-known pathological progression of AD (Braak and Braak, 1991; Frisoni et al., 2010; Jucker and Walker, 2011), as well as the distribution of structural atrophy in MCI-converters relative to non-converters: baseline atrophy in medial temporal structures including the entorhinal cortex (Tapiola et al., 2008), hippocampus (Tapiola et al., 2008; Risacher et al., 2009), posterior cingulate gyrus (Querbes et al., 2009), and left temporal lobe (Ferreira et al., 2011). Additionally, the “yellow” and “black” modules were involved in regions that exhibited baseline differences in cortical thickness between AD-like patterned MCI brains and normal-like patterned MCI brains, specifically in the entorhinal cortex, fusiform gyrus, temporal pole, superior temporal gyrus, middle temporal gyrus, and inferior temporal gyrus (Falahati et al., 2017). Our results were consistent with these earlier studies, which support our hypothesis that this data-driven network approach originating from genetics can detect hidden structural atrophies.

Although the WGCNA approach is advantageous due to its data-driven manner, there have been several studies using data-driven multivariate analyses such as independent component analysis (ICA) (Iraji et al., 2016) or principle component analysis (PCA) (Leonardi et al., 2013) to investigate connectivity among brain regions in AD or MCI. To illustrate, the decreasing functional brain connectivity in MCI and AD along with cognitive decline progression has been reported in an ICA-based fMRI study (Pagani et al., 2017). The WGCNA has a number of advantages in comparison with these approaches, as the ICA requires spatial or temporal independence assumption (Iraji et al., 2016), which may not be plausible for complexed and interconnected pathological changes among brain regions, while the PCA assumes Gaussian distribution of the data. Additionally, the WVCNA study on fMRI demonstrated that this method tends to produce more spatially focused modules than that of ICA components (Mumford et al., 2010).

Since this method could detect previously reported underlying pathological extension of AD, using only the structural images in an unsupervised manner, radiological studies of other neurodegenerative diseases may also benefit from this method. We can further evaluate the validity of this method in other neurodegenerative diseases which are pathologically well investigated such as Lewy body disease. Consequently, we may be able to obtain informative suggestions to estimate or interpret the pathological progression in other neurodegenerative disease that are much rarer and have not been thoroughly investigated pathologically, such as neuronal intranuclear hyaline inclusion disease.

Another advantage in using WGCNA is that we calculate the first principle component of the module, giving us the opportunity to speculate over its characteristics in association with the clinical features. To illustrate, considering age, several modules have significant correlation with age specifically that cortical atrophy and ventricular enlargement was associated with baseline age (Fig. 4, leftmost column); however, these correlations become non-significant in the following 36 months (Fig. 5, leftmost column), suggesting that atrophy in these cortices was not always accelerated by a greater baseline age. A similar relationship is observed between the degree of regional atrophy and baseline renal function (Fig. 4, Fig. 5, third column from the right). Additionally, while the baseline positivity of amyloid-PET or *APOE* allele status was not associated with most modules at baseline in MCI participants (Fig. 4, fourth and seventh columns from the right, respectively), these features are significantly associated with the salmon module (containing bilateral lateral ventricles), tan module (containing bilateral ventral diencephalons), purple module (containing bilateral caudate nuclei), brown module, or yellow module at the 36 month follow-up. This suggests that these AD pathology-related features have an effect on the regional/structural MRI changes in a delayed manner (for up to 36 months). A similar delay can also be observed between baseline CSF biomarkers and enlargement of the lateral ventricles.

Our study has certain limitations. First, is the restrictions associated with the WGCNA methodological requirements, in which the data structure should be equipped with unified features (e.g., anatomical standardization in voxel-based morphometry) and data measurements be normalized. This is sometimes time-consuming if using data from *FreeSurfer* and the process to define a reference in dataset normalization can be another concern. Furthermore, our analysis did not consider similarities between anatomically neighboring regions/structures, which should demonstrate a high correlation in the pathological involvement. It should also be noted that the atrophy of entorhinal cortex, hippocampus, or amygdala, have significant associations with clinical deterioration in MCI but were not included in any of these modules in the present result. This is presumably due to the similarities in their regions/structures', implying that this methodology does not always cover all individual regional/structural associations with the given clinical features. Additionally, modularization results can differ depending on the adjustment of the parameters, thereby making this method less statistically robust. Although we considered both cortical thinning and volume reduction as regional/structural atrophy, the significance of these two is not always equivalent.

## 5 Conclusions

To conclude, we quantitatively visualized coordinated distribution and progression of brain atrophy following AD progression using the WGCNA methodology on the J-ADNI cohort's structural brain MRI data for MCI participants, and largely demonstrated results similar to earlier AD/MCI studies. Our results suggest the potential applicability of this methodology as an option to visualize underlying pathological progression in integrative large-scale connectivity studies of neurodegenerative disease, not just limited to AD.

The following are the supplementary data related to this article.

Supplementary material 1. Co-investigator appendix Image 1

Supplementary material 2. Supplemental Table 1-3 Image 2

Supplementary material 3. Summary of FreeSurfer data of each region/structure at baseline and 36 months. Image 3

## Conflicts of interest

The authors have no conflict of interest to disclose.

## Ethical approval

The study protocol was approved by the University of Tokyo ethics committee (11628).
