# Supplementary material for: Visualizing modules of coordinated structural brain atrophy during the course of conversion to Alzheimer's disease by applying methodology from gene co-expression analysis
Source: Neuroimage Clin. 2019 Jul 25;24:101957. doi: 10.1016/j.nicl.2019.101957 (PMC6700430; doi:10.1016/j.nicl.2019.101957)
Supplement: Supplementary file 1 — Supplementary material 1. Co-investigator appendix [file mmc1.docx]

**Co-investigator appendix**

Takeshi Iwatsubo (The University of Tokyo, PI of J-ADNI), Takashi Asada (Tsukuba University, Clinical Core Leaders), Hiroyuki Arai (Tohoku University, Clinical Core Leaders and Biochemistry Core Leaders), Morihiro Sugishita (Institute of Brain and Blood Vessels, Clinical Core Leaders), Hiroshi Matsuda (National Center of Neurology and Psychiary, MRI Core Leaders and IT Core Leaders, Saitama Medical University, Site Investigator), Kengo Ito (National Center of Geriatrics and Gerontology, PET Core Leaders), Michio Senda (Institute of Biomedical Research and Innovation, PET Core Leaders), Kenji Ishii (Tokyo Metropolitan Geriatric Hospital and Institute of Gerontology, PET Core Leaders), Ryozo Kuwano (Niigata University, Biochemistry Core Leaders), Takeshi Ikeuchi (Niigata University, Biochemistry Core Leaders), Noriko Sato (National Center of Neurology and Psychiary, IT Core Leaders), Hajime Sato (University of Tokyo, Statistics Core Leaders), Shun Shimohama (Sapporo Medical University Hospital, Site Investigator), Masaki Saitoh (Sapporo Medical University Hospital, Site Investigator), Rika Yamauchi (Sapporo Medical University Hospital, Site Investigator), Takashi Hayashi (Sapporo Medical University Hospital, Site Investigator), Seiju Kobayashi (Sapporo Medical University Hospital, Site Investigator), Norihito Nakano (University of Hokkaido, Site Investigator), Junichiro Kanazawa (University of Hokkaido, Site Investigator), Takeshi Ando (University of Hokkaido, Site Investigator), Chiyoko Takanami (Sapporo Medical University, Site Investigator), Masato Hareyama (Sapporo Medical University Hospital, Site Investigator), Masamitsu Hatakenaka (Sapporo Medical University Hospital, Site Investigator), Eriko Tsukamoto (Social Medical Corporation Teishinkai Central CI Clinic, Site Investigator), Shinji Ochi (Social Medical Corporation Teishinkai Central CI Clinic, Site Investigator), Mikio Shoji (Hirosaki University, Site Investigator), Etsuro Matsubara (Hirosaki University, Site Investigator), Takeshi Kawarabayashi (Hirosaki University, Site Investigator), Yasuhito Wakasaya (Hirosaki University, Site Investigator), Takashi Nakata (Hirosaki University, Site Investigator), Naoko Nakahata (Hirosaki University, Site Investigator), Shuichi Ono (Hirosaki University, Site Investigator), Yoshihiro Takai (Hirosaki University, Site Investigator), Satoshi Takahashi (Iwate Medical University, Site Investigator), Hisashi Yonezawa (Iwate Medical University, Site Investigator), Junko Takahashi (Iwate Medical University, Site Investigator), Masako Kudoh (Iwate Medical University, Site Investigator), Makoto Sasaki (Iwate Medical University, Site Investigator), Yutaka Matsumura (Iwate Medical University, Site Investigator), Yohsuke Hirata (Iwate Medical University, Site Investigator), Tsuyoshi Metoki (Iwate Medical University, Site Investigator), Susumu Hayakawa (Iwate Medical University, Site Investigator), Yuichi Sato (Iwate Medical University, Site Investigator), Masayuki Takeda (Iwate Medical University, Site Investigator), Toshiaki Sasaki (Iwate Medical University, Site Investigator), Koichiro Sera (Iwate Medical University, Site Investigator), Kazunori Terasaki (Iwate Medical University, Site Investigator), Yoshihiro Saitoh (Nishina Memorial Cyclotron Center, Japan Radioisotope Association, Site Investigator), Shoko Goto(Nishina Memorial Cyclotron Center, Japan Radioisotope Association, Site Investigator), Kuniko Ueno (Iwate Medical University, Site Investigator), Hiromi Sakashita (Iwate Medical University, Site Investigator), Kuniko Watanabe (Iwate Medical University, Site Investigator), Ken Nagata (Research Institute for Brain and Blood Vessels-Akita, Site Investigator), Yuichi Sato (Research Institute for Brain and Blood Vessels-Akita, Site Investigator), Tetsuya Maeda (Research Institute for Brain and Blood Vessels-Akita, Site Investigator), Yasushi Kondoh (Research Institute for Brain and Blood Vessels-Akita, Site Investigator), Takashi Yamazaki (Research Institute for Brain and Blood Vessels-Akita, Site Investigator), Daiki Takano (Research Institute for Brain and Blood Vessels-Akita, Site Investigator), Mio Miyata (Research Institute for Brain and Blood Vessels-Akita, Site Investigator), Hiromi Komatsu (Research Institute for Brain and Blood Vessels-Akita, Site Investigator), Mayumi Watanabe (Research Institute for Brain and Blood Vessels-Akita, Site Investigator), Tomomi Shinoda (Research Institute for Brain and Blood Vessels-Akita, Site Investigator), Rena Muraoka (Research Institute for Brain and Blood Vessels-Akita, Site Investigator), Kayoko Kikuchi (Research Institute for Brain and Blood Vessels-Akita, Site Investigator), Hitomi Ito (Research Institute for Brain and Blood Vessels-Akita, Site Investigator), Aki Sato (Research Institute for Brain and Blood Vessels-Akita, Site Investigator), Toshibumi Kinoshita (Research Institute for Brain and Blood Vessels-Akita, Site Investigator), Hideyo Toyoshima (Research Institute for Brain and Blood Vessels-Akita, Site Investigator), Kaoru Sato (Research Institute for Brain and Blood Vessels-Akita, Site Investigator), Shigeki Sugawara (Research Institute for Brain and Blood Vessels-Akita, Site Investigator), Isao Ito (Research Institute for Brain and Blood Vessels-Akita, Site Investigator), Fumiko Kumagai (Research Institute for Brain and Blood Vessels-Akita, Site Investigator), Katsutoshi Furukawa (Tohoku University, Site Investigator), Masaaki Waragai (Tohoku University, Site Investigator), Naoki Tomita (Tohoku University, Site Investigator), Nobuyuki Okamura (Tohoku University, Site Investigator), Mari Ootsuki (Tohoku University, Site Investigator), Katsumi Sugawara (Tohoku University, Site Investigator), Satomi Sugawara (Tohoku University, Site Investigator), Shunji Mugikura (Tohoku University, Site Investigator), Atsushi Umetsu (Tohoku University, Site Investigator), Takanori Murata (Tohoku University, Site Investigator), Tatsuo Nagasaka (Tohoku University, Site Investigator), Yukitsuka Kudo (Tohoku University, Site Investigator), Manabu Tashiro (Tohoku University, Site Investigator), Shoichi Watanuki (Tohoku University, Site Investigator), Masatoyo Nishizawa (Niigata University, Site Investigator), Takeshi Ikeuchi (Niigata University, Site Investigator), Takayoshi Tokutake (Niigata University, Site Investigator), Saeri Ishikawa (Niigata University, Site Investigator), Emiko Kishida (Niigata University, Site Investigator), Nozomi Sato (Niigata University, Site Investigator), Mieko Hagiwara (Niigata University, Site Investigator), Kumi Yamanaka (Niigata University, Site Investigator), Takeyuki Watanabe (Niigata University, Site Investigator), Taeko Takasugi (Niigata University, Site Investigator), Shoichi Inagawa (Niigata University, Site Investigator), Kenichi Naito (Niigata University, Site Investigator), Masanori Awaji (Niigata University, Site Investigator), Tsutomu Kanazawa (Niigata University, Site Investigator), Kouiti Okamoto (Gunma University, Site Investigator), Masaki Ikeda (Gunma University, Site Investigator), Tsuneo Yamazaki (Gunma University, Site Investigator), Yuichi Tashiro (Gunma University, Site Investigator), Shun Nagamine (Gunma University, Site Investigator), Shiori Katsuyama (Gunma University, Site Investigator), Sachiko Kurose (Gunma University, Site Investigator), Sayuri Fukushima (Gunma University, Site Investigator), Etsuko Koya (Gunma University, Site Investigator), Makoto Amanuma (Gunma University, Site Investigator), Noboru Oriuchi (Gunma University, Site Investigator), Koichi Ujita (Gunma University, Site Investigator), Kazuhiro Kishi (Gunma University, Site Investigator), Kazuhisa Tsuda (Gunma University, Site Investigator), Katsuyoshi Mizukami (Tsukuba University, Site Investigator), Tetsuaki Arai (Tsukuba University, Site Investigator), Etsuko Nakajima (Tsukuba University, Site Investigator), Katsumi Miyamoto (Tsukuba Medical Center Hospital, Site Investigator), Kousaku Saotome (Tsukuba Medical Center Hospital, Site Investigator), Tomoya Kobayashi (Tsukuba Medical Center Hospital, Site Investigator), Saori Itoya (Tsukuba Medical Center Hospital, Site Investigator), Jun Ookubo (Tsukuba Medical Center Hospital, Site Investigator), Toshiya Akatsu (Tsukuba Medical Center Hospital, Site Investigator), Yoshiko Anzai (Tsukuba Medical Center Hospital, Site Investigator), Junya Ikegaki (Tsukuba Medical Center Hospital, Site Investigator), Yuuichi Katou (Tsukuba Medical Center Hospital, Site Investigator), Kaori Kimura (Tsukuba Medical Center Hospital, Site Investigator), Ryou Kuchii (Tsukuba Medical Center Hospital, Site Investigator), Hajime Saitou (Tsukuba Medical Center Hospital, Site Investigator), Kazuya Shinoda (Tsukuba Medical Center Hospital, Site Investigator), Satoka Someya (Tsukuba Medical Center Hospital, Site Investigator), Hiroko Taguchi (Tsukuba Medical Center Hospital, Site Investigator), Kazuya Tashiro (Tsukuba Medical Center Hospital, Site Investigator), Masaya Tanaka (Tsukuba Medical Center Hospital, Site Investigator), Tatsuya Nemoto (Tsukuba Medical Center Hospital, Site Investigator), Ryou Wakabayashi (Tsukuba Medical Center Hospital, Site Investigator), Daisuke Watanabe (Tsukuba Medical Center Hospital, Site Investigator), Harumasa Takano (National Institute of Radiological Sciences, Site Investigator), Tetsuya Suhara (National Institute of Radiological Sciences, Site Investigator), Hitoshi Shinotoh (National Institute of Radiological Sciences, Site Investigator, Asahi Hospital for Neurological Diseases and Rehabilitation, Site Investigator), Hitoshi Shimada (National Institute of Radiological Sciences, Site Investigator, Asahi Hospital for Neurological Diseases and Rehabilitation, Site Investigator), Makoto Higuchi (National Institute of Radiological Sciences, Site Investigator), Takaaki Mori (National Institute of Radiological Sciences, Site Investigator), Hiroshi Ito (National Institute of Radiological Sciences, Site Investigator), Takayuki Obata (National Institute of Radiological Sciences, Site Investigator), Yoshiko Fukushima (National Institute of Radiological Sciences, Site Investigator), Kazuko Suzuki (National Institute of Radiological Sciences, Site Investigator), Izumi Izumida (National Institute of Radiological Sciences, Site Investigator), Katsuyuki Tanimoto (National Institute of Radiological Sciences, Site Investigator), Takahiro Shiraishi (National Institute of Radiological Sciences, Site Investigator), Junko Shiba (Asahi Hospital for Neurological Diseases and Rehabilitation, Site Investigator), Hiroaki Yano (Asahi Hospital for Neurological Diseases and Rehabilitation, Site Investigator), Miki Satake (Asahi Hospital for Neurological Diseases and Rehabilitation, Site Investigator), Aimi Nakui (Asahi Hospital for Neurological Diseases and Rehabilitation, Site Investigator), Yae Ebihara (Asahi Hospital for Neurological Diseases and Rehabilitation, Site Investigator), Tomomi Hasegawa (Asahi Hospital for Neurological Diseases and Rehabilitation, Site Investigator), Yasumasa Yoshiyama (National Hospital Organization Chiba-East-Hospital, Site Investigator), Mami Kato (National Hospital Organization Chiba-East-Hospital, Site Investigator), Yuki Ogata (National Hospital Organization Chiba-East-Hospital, Site Investigator), Hiroyuki Fujikawa (National Hospital Organization Chiba-East-Hospital, Site Investigator), Nobuo Araki (Saitama Medical University, Site Investigator), Yoshihiko Nakazato (Saitama Medical University, Site Investigator), Takahiro Sasaki (Saitama Medical University, Site Investigator), Tomokazu Shimazu (Saitama Medical University, Site Investigator), Kimiko Yoshimaru (Saitama Medical University, Site Investigator), Etsuko Imabayashi (Saitama Medical University, Site Investigator), Asako Yasuda (Saitama Medical University, Site Investigator), Etsuko Yamamoto (Saitama Medical University, Site Investigator), Natsumi Nakamata (Saitama Medical University, Site Investigator), Noriko Miyauchi (Saitama Medical University, Site Investigator), Keiko Ozawa (Saitama Medical University, Site Investigator), Rieko Hashimoto (Saitama Medical University, Site Investigator), Taishi Unezawa (Saitama Medical University, Site Investigator), Takafumi Ichikawa (Saitama Medical University, Site Investigator), Hiroki Hayashi (Saitama Medical University, Site Investigator), Masakazu Yamagishi (Saitama Medical University, Site Investigator), Tsunemichi Mihara (Saitama Medical University, Site Investigator), Masaya Hirano (Saitama Medical University, Site Investigator), Shinichi Watanabe (Saitama Medical University, Site Investigator), Junichiro Fukuhara (Saitama Medical University, Site Investigator), Hajime Matsudo (Saitama Medical University, Site Investigator), Nobuyuki Saito (SHI Accelerator Service Ltd.(SAS) , Site Investigator), Atsushi Iwata (The University of Tokyo, Site Investigator), Hisatomo Kowa (The University of Tokyo, Site Investigator), Toshihiro Hayashi (The University of Tokyo, Site Investigator), Ryoko Ihara (The University of Tokyo, Site Investigator), Toji Miyagawa (The University of Tokyo, Site Investigator), Mizuho Yoshida (The University of Tokyo, Site Investigator), Yuri Koide (The University of Tokyo, Site Investigator), Eriko Samura (The University of Tokyo, Site Investigator), Kurumi Fujii (The University of Tokyo, Site Investigator), Kaori Watanabe (The University of Tokyo, Site Investigator), Nagae Orihara (The University of Tokyo, Site Investigator), Toshimitsu Momose (The University of Tokyo, Site Investigator), Akira Kunimatsu (The University of Tokyo, Site Investigator), Harushi Mori (The University of Tokyo, Site Investigator), Miwako Takahashi (The University of Tokyo, Site Investigator), Takuya Arai (The University of Tokyo, Site Investigator), Yoshiki Kojima (The University of Tokyo, Site Investigator), Masami Goto (The University of Tokyo, Site Investigator), Takeo Sarashina (The University of Tokyo, Site Investigator), Syuichi Uzuki (The University of Tokyo, Site Investigator), Seiji Katou (The University of Tokyo, Site Investigator), Yoshiharu Sekine (The University of Tokyo, Site Investigator), Yukihiro Takauchi (The University of Tokyo, Site Investigator), Chiine Kagami (The University of Tokyo, Site Investigator), Kazutomi Kanemaru (Tokyo Metropolitan Geriatric Hospital and Institute of Gerontology, Site Investigator), Shigeo Murayama (Tokyo Metropolitan Geriatric Hospital and Institute of Gerontology, Site Investigator), Yasushi Nishina (Tokyo Metropolitan Geriatric Hospital and Institute of Gerontology, Site Investigator), Kenji Ishii (Tokyo Metropolitan Geriatric Hospital and Institute of Gerontology, Site Investigator), Maria Sakaibara (Tokyo Metropolitan Geriatric Hospital and Institute of Gerontology, Site Investigator), Yumiko Okazaki (Tokyo Metropolitan Geriatric Hospital and Institute of Gerontology, Site Investigator), Rieko Okada (Tokyo Metropolitan Geriatric Hospital and Institute of Gerontology, Site Investigator), Maki Obata (Tokyo Metropolitan Geriatric Hospital and Institute of Gerontology, Site Investigator), Yuko Iwata (Clinical Support Corporation, Site Investigator), Mizuho Minami (Clinical Support Corporation, Site Investigator), Yasuko Hanabusa (Clinical Support Corporation, Site Investigator), Hanae Shingyouji (Clinical Support Corporation, Site Investigator), Kyoko Tottori (Clinical Support Corporation, Site Investigator), Aya Tokumaru (Tokyo Metropolitan Geriatric Hospital and Institute of Gerontology, Site Investigator), Makoto Ichinose (Tokyo Metropolitan Geriatric Hospital and Institute of Gerontology, Site Investigator), Kazuya Kume (Tokyo Metropolitan Geriatric Hospital and Institute of Gerontology, Site Investigator), Shunsuke Kahashi (Tokyo Metropolitan Geriatric Hospital and Institute of Gerontology, Site Investigator), Kunimasa Arima (National Center Hospital, National Center of Neurology and Psychiatry, Site Investigator), Tadashi Tsukamoto (National Center Hospital, National Center of Neurology and Psychiatry, Site Investigator), Shin Tanaka (National Center Hospital, National Center of Neurology and Psychiatry, Site Investigator), Yuko Nagahusa (National Center Hospital, National Center of Neurology and Psychiatry, Site Investigator), Masuhiro Sakata (National Center Hospital, National Center of Neurology and Psychiatry, Site Investigator), Mitsutoshi Okazaki (National Center Hospital, National Center of Neurology and Psychiatry, Site Investigator), Yuko Saito (National Center Hospital, National Center of Neurology and Psychiatry, Site Investigator), Maki Yamada (National Center Hospital, National Center of Neurology and Psychiatry, Site Investigator), Chiine Kodama (National Center Hospital, National Center of Neurology and Psychiatry, Site Investigator), Maki Obata (National Center Hospital, National Center of Neurology and Psychiatry, Site Investigator), Tomoko Takeuchi (National Center Hospital, National Center of Neurology and Psychiatry, Site Investigator), Keiichiro Ozawa (National Center Hospital, National Center of Neurology and Psychiatry, Site Investigator), Yoshiko Kawaji (Clinical Support Corporation, Site Investigator), Noriko Sato (National Center Hospital, National Center of Neurology and Psychiatry, Site Investigator), Yasuhiro Nakata (National Center Hospital, National Center of Neurology and Psychiatry, Site Investigator), Satoshi Sawada (National Center Hospital, National Center of Neurology and Psychiatry, Site Investigator), Makoto Mimatsu (National Center Hospital, National Center of Neurology and Psychiatry, Site Investigator), Daisuke Nakkamura (National Center Hospital, National Center of Neurology and Psychiatry, Site Investigator), Takeshi Tamaru (National Center Hospital, National Center of Neurology and Psychiatry, Site Investigator), Shunichirou Horiuchi (National Center Hospital, National Center of Neurology and Psychiatry, Site Investigator), Heii Arai (Juntendo University, Site Investigator), Tsuneyoshi Ota (Juntendo University, Site Investigator), Aiko Kodaka (Juntendo University, Site Investigator), Yuko Tagata (Juntendo University, Site Investigator), Tomoko Nakada (Juntendo University, Site Investigator), Aiko Kodaka (Juntendo University, Site Investigator), Yuko Tagata (Juntendo University, Site Investigator), Tomoko Nakada (Juntendo University, Site Investigator), Eizo Iseki (Juntendo Tokyo Koto Geriatric Medical Center, Site Investigator), Kiyoshi Sato (Juntendo Tokyo Koto Geriatric Medical Center, Site Investigator), Hiroshige Fujishiro (Juntendo Tokyo Koto Geriatric Medical Center, Site Investigator), Norio Murayama (Juntendo Tokyo Koto Geriatric Medical Center, Site Investigator), Kiyoshi Sato (Juntendo Tokyo Koto Geriatric Medical Center, Site Investigator), Masaru Suzuki (Juntendo Tokyo Koto Geriatric Medical Center, Site Investigator), Satoshi Kimura (Juntendo Tokyo Koto Geriatric Medical Center, Site Investigator), Masanobu Takahashi (Juntendo Tokyo Koto Geriatric Medical Center, Site Investigator), Haruo Hanyu (Tokyo Medical University, Site Investigator), Hirofumi Sakurai (Tokyo Medical University, Site Investigator), Takahiko Umahara (Tokyo Medical University, Site Investigator), Hidekazu Kanetaka (Tokyo Medical University, Site Investigator), Kaori Arashino (Tokyo Medical University, Site Investigator), Mikako Murakami (Tokyo Medical University, Site Investigator), Ai Kito (Tokyo Medical University, Site Investigator), Seiko Miyagi (Tokyo Medical University, Site Investigator), Kaori Doi (Tokyo Medical University, Site Investigator), Kazuyoshi Sasaki (Tokyo Medical University, Site Investigator), Mineo Yamazaki (Nippon Medical School, Site Investigator), Akiko Ishiwata (Nippon Medical School, Site Investigator), Yasushi Arai (Nippon Medical School, Site Investigator), Akane Nogami (Nippon Medical School, Site Investigator), Sumiko Fukuda (Nippon Medical School, Site Investigator), Koichi Kozaki (Kyorin University, Site Investigator), Yukiko Yamada (Kyorin University, Site Investigator), Sayaka Kimura (Kyorin University, Site Investigator), Ayako Machida (Kyorin University, Site Investigator), Kuninori Kobayashi (Kyorin University, Site Investigator), Hidehiro Mizusawa (Tokyo Medical and Dental University, Site Investigator), Nobuo Sanjo (Tokyo Medical and Dental University, Site Investigator), Mutsufusa Watanabe (Tokyo Medical and Dental University, Site Investigator), Takuya Ohkubo (Tokyo Medical and Dental University, Site Investigator), Hiromi Utashiro (Tokyo Medical and Dental University, Site Investigator), Yukiko Matsumoto (Tokyo Medical and Dental University, Site Investigator), Kumiko Hagiya (Tokyo Medical and Dental University, Site Investigator), Yoshiko Miyama (Tokyo Medical and Dental University, Site Investigator), Takako Shinozaki (Tokyo Medical and Dental University, Site Investigator), Haruko Hiraki (Tokyo Medical and Dental University, Site Investigator), Hitoshi Shibuya (Tokyo Medical and Dental University, Site Investigator), Isamu Ohashi (Tokyo Medical and Dental University, Site Investigator), Akira Toriihara (Tokyo Medical and Dental University, Site Investigator), Shinichi Ohtani (Tokyo Medical and Dental University, Site Investigator), Toshifumi Matsui (National Hospital Organization, Kurihama Medical & Addiction Center, Site Investigator), Yu Hayasaka (Kitasato University, Site Investigator), Tomomi Toyama (National Hospital Organization, Kurihama Medical & Addiction Center, Site Investigator), Hideki Sakurai (National Hospital Organization, Kurihama Medical & Addiction Center, Site Investigator), Kumiko Sugiura (National Hospital Organization, Kurihama Medical & Addiction Center, Site Investigator), Hirofumi Taguchi (National Hospital Organization, Kurihama Medical & Addiction Center, Site Investigator), Shizuo Hatashita (Shonan-Atsugi Hospital, Site Investigator), Akari Imuta (Shonan-Atsugi Hospital, Site Investigator), Akiko Matsudo (Shonan-Atsugi Hospital, Site Investigator), Daichi Wakebe (Shonan-Atsugi Hospital, Site Investigator), Hideki Hayakawa (Shonan-Atsugi Hospital, Site Investigator), Mitsuhiro Ono (Shonan-Atsugi Hospital, Site Investigator), Takayoshi Ohara (Shonan-Atsugi Hospital, Site Investigator), Yukihiko Washimi (National Center of Geriatrics and Gerontology, Site Investigator), Yutaka Arahata (National Center of Geriatrics and Gerontology, Site Investigator), Akinori Takeda (National Center of Geriatrics and Gerontology, Site Investigator), Yoko Konagaya (National Center of Geriatrics and Gerontology, Site Investigator), Akiko Yamaoka (National Center of Geriatrics and Gerontology, Site Investigator), Masashi Tsujimoto (National Center of Geriatrics and Gerontology, Site Investigator), Hideyuki Hattori (National Center of Geriatrics and Gerontology, Site Investigator), Takashi Sakurai (National Center of Geriatrics and Gerontology, Site Investigator), Miura Hisayuki (National Center of Geriatrics and Gerontology, Site Investigator), Hidetoshi Endo (National Center of Geriatrics and Gerontology, Site Investigator), Syousuke Satake (National Center of Geriatrics and Gerontology, Site Investigator), Young Jae Hong (National Center of Geriatrics and Gerontology, Site Investigator), Katsunari Iwai (National Center of Geriatrics and Gerontology, Site Investigator), Kenji Yoshiyama (National Center of Geriatrics and Gerontology, Site Investigator), Masaki Suenaga (National Center of Geriatrics and Gerontology, Site Investigator), Sumiko Morita (National Center of Geriatrics and Gerontology, Site Investigator), Teruhiko Kachi (National Center of Geriatrics and Gerontology, Site Investigator), Kenji Toba (National Center of Geriatrics and Gerontology, Site Investigator), Rina Miura (National Center of Geriatrics and Gerontology, Site Investigator), Takiko Kawai (National Center of Geriatrics and Gerontology, Site Investigator), Ai Honda (National Center of Geriatrics and Gerontology, Site Investigator), Kengo Ito (National Center of Geriatrics and Gerontology, Site Investigator), Takashi Kato (National Center of Geriatrics and Gerontology, Site Investigator), Ken Fujiwara (National Center of Geriatrics and Gerontology, Site Investigator), Rikio Kato (National Center of Geriatrics and Gerontology, Site Investigator), Mariko Koyama (National Center of Geriatrics and Gerontology, Site Investigator), Naohiko Fukaya (National Center of Geriatrics and Gerontology, Site Investigator), Akira Tsuji (National Center of Geriatrics and Gerontology, Site Investigator), Hitomi Shimizu (National Center of Geriatrics and Gerontology, Site Investigator), Hiroyuki Fujisawa (National Center of Geriatrics and Gerontology, Site Investigator), Tomoko Nakazawa (National Center of Geriatrics and Gerontology, Site Investigator), Satoshi Koyama (National Center of Geriatrics and Gerontology, Site Investigator), Takanori Sakata (National Center of Geriatrics and Gerontology, Site Investigator), Masahito Yamada (Kanazawa University, Site Investigator), Mitsuhiro Yoshita (Kanazawa University, Site Investigator), Miharu Samuraki (Kanazawa University, Site Investigator), Kenjiro Ono (Kanazawa University, Site Investigator), Moeko Shinohara (Kanazawa University, Site Investigator), Yuki Soshi (Kanazawa University, Site Investigator), Kozue Niwa (Kanazawa University, Site Investigator), Chiaki Doumoto (Kanazawa University, Site Investigator), Mariko Hata (Kanazawa University, Site Investigator), Miyuki Matsushita (Kanazawa University, Site Investigator), Mai Tsukiyama (Kanazawa University, Site Investigator), Nozomi Takeda (The Medical and Pharmacological Rearch Center Foundation, Site Investigator), Sachiko Yonezawa (The Medical and Pharmacological Rearch Center Foundation, Site Investigator), Ichiro Matsunari (The Medical and Pharmacological Rearch Center Foundation, Site Investigator), Osamu Matsui (Kanazawa University, Site Investigator), Fumiaki Ueda (Kanazawa University, Site Investigator), Yasuji Ryu (Kanazawa University, Site Investigator), Masanobu Sakamoto (Hamamatsu Medical Center, Site Investigator), Yasuomi Ouchi (Hamamatsu Medical Center, Site Investigator), Madoka Chita (Hamamatsu Medical Center, Site Investigator), Yumiko Fujita (Hamamatsu Medical Center, Site Investigator), Rika Majima (Hamamatsu Medical Center, Site Investigator), Hiromi Tsubota (Hamamatsu Medical Center, Site Investigator), Umeo Shirasawa (Hamamatsu Medical Center, Site Investigator), Masashi Sugimori (Hamamatsu Medical Center, Site Investigator), Wataru Ariya (Hamamatsu Medical Center, Site Investigator), Yuuzou Hagiwara (Hamamatsu Medical Center, Site Investigator), Yasuo Tanizaki (Hamamatsu Medical Center, Site Investigator), Hidenao Fukuyama (Kyoto University, Site Investigator), Ryosuke Takahashi (Kyoto University, Site Investigator), Hajime Takechi (Kyoto University, Site Investigator), Chihiro Namiki (Eli Lilly Japan K.K Medical Science, Site Investigator), Kengo Uemura (Kyoto University, Site Investigator), Takeshi Kihara (Rakuwakai Misasagi Hospital, Site Investigator), Hiroshi Yamauchi (Shiga Medical Center Research Institute, Site Investigator), Shizuko Tanaka-Urayama (Kyoto University, Site Investigator), Emiko Maeda (Takemura Clinic, Site Investigator), Natsu Saito (Tonami General Hospital, Site Investigator), Shiho Satomi (Kyoto Prefectural University of Medicine, Site Investigator), Konomi Kabata (Kyoto University, Site Investigator), Shin-ichi Urayama (Kyoto University, Site Investigator), Tomohisa Okada (Kyoto University, Site Investigator), Koichi Ishizu (Kyoto University, Site Investigator), Shigeto Kawase (Kyoto University, Site Investigator), Satoshi Fukumoto (Kyoto University, Site Investigator), Masanori Nakagawa (Kyoto Prefectural University of Medicine, Site Investigator), Takahiko Tokuda (Kyoto Prefectural University of Medicine, Site Investigator), Masaki Kondo (Kyoto Prefectural University of Medicine, Site Investigator), Fumitoshi Niwa (Kyoto Prefectural University of Medicine, Site Investigator), Toshiki Mizuno (Kyoto Prefectural University of Medicine, Site Investigator), Yoko Oishi (Kyoto Prefectural University of Medicine, Site Investigator), Mariko Yamazaki (Kyoto Prefectural University of Medicine, Site Investigator), Daisuke Yamaguchi (Kyoto Prefectural University of Medicine, Site Investigator), Kyoko Ito (Tokyo Yakuriken Co.,Ltd., Site Investigator), Yoku Asano (Tokyo Yakuriken Co.,Ltd., Site Investigator), Chizuru Hamaguchi (Tokyo Yakuriken Co.,Ltd., Site Investigator), Kei Yamada (Kyoto Prefectural University of Medicine, Site Investigator), Chio Okuyama (Kyoto Prefectural University of Medicine, Site Investigator), Kentaro Akazawa (Kyoto Prefectural University of Medicine, Site Investigator), Shigenori Matsushima (Kyoto Prefectural University of Medicine, Site Investigator), Takamasa Matsuo (Kyoto Prefectural University of Medicine, Site Investigator), Toshiaki Nakagawa (Kyoto Prefectural University of Medicine, Site Investigator), Takeshi Nii (Kyoto Prefectural University of Medicine, Site Investigator), Takuji Nishida (Kyoto Prefectural University of Medicine, Site Investigator), Kuniaki Kiuchi (Nara Medical University, Site Investigator), Masami Fukusumi (Doshisha University, Site Investigator), Hideyuki Watanabe (Heartland Shigisan Hospital, Site Investigator), Toshiaki Taoka (Nara Medical University, Site Investigator), Akihiro Nogi (Nara Medical University, Site Investigator), Masatoshi Takeda (Osaka University, Site Investigator), Toshihisa Tanaka (Osaka University, Site Investigator), Naoyuki Sato (Osaka University, Site Investigator), Hiroaki Kazui (Osaka University, Site Investigator), Kenji Yoshiyama (Osaka University, Site Investigator), Takashi Kudo (Osaka University, Site Investigator), Masayasu Okochi (Osaka University, Site Investigator), Takashi Morihara (Osaka University, Site Investigator), Shinji Tagami (Osaka University, Site Investigator), Noriyuki Hayashi (Osaka University, Site Investigator), Masahiko Takaya (Osaka University, Site Investigator), Tamiki Wada (Osaka University, Site Investigator), Mikiko Yokokoji (Osaka University, Site Investigator), Hiromichi Sugiyama (Osaka University, Site Investigator), Daisuke Yamamoto (Osaka University, Site Investigator), Shuko Takeda (Japan Society for the Promotion of Science, Site Investigator), Keiko Nomura (Osaka University, Site Investigator), Mutsumi Tomioka (Osaka University, Site Investigator), Eiichi Uchida (Uchida Clinic, Site Investigator), Yoshiyuki Ikeda (Uchida Clinic, Site Investigator), Mineto Murakami (Uchida Clinic, Site Investigator), Takami Miki (Osaka City University, Site Investigator), Hiroyuki Shimada (Osaka City University, Site Investigator), Suzuka Ataka (Osaka City University, Site Investigator), Motokatsu Kanemoto (Osaka Municipal Kohsaiin Hospital, Site Investigator), Jun Takeuchi (Kishiwada City Hospital, Site Investigator), Akitoshi Takeda (Osaka City University, Site Investigator), Rie Azuma (Kosaka Hospital, Site Investigator), Yuki Iwamoto (Osaka City University, Site Investigator), Naomi Tagawa (Osaka City University, Site Investigator), Junko Masao (Osaka City University, Site Investigator), Yuka Matsumoto (Osaka City University, Site Investigator), Yuko Kikukawa (Osaka City University, Site Investigator), Hisako Fujii (Osaka City University, Site Investigator), Junko Matsumura (Osaka City University, Site Investigator), Susumu Shiomi (Osaka City University, Site Investigator), Joji Kawabe (Osaka City University, Site Investigator), Yoshihiro Shimonishi (Osaka City University, Site Investigator), Yukio Miki (Osaka City University, Site Investigator), Mitsuji Higashida (Osaka City University, Site Investigator), Tomohiro Sahara (Osaka City University, Site Investigator), Takashi Yamanaga (Osaka City University, Site Investigator), Shinichi Sakamoto (Osaka City University, Site Investigator), Hiroyuki Tsushima (Ibaraki Prefectural University of Health Sciences, Site Investigator), Kiyoshi Maeda (Kobe University, Site Investigator), Yasuji Yamamoto (Kobe University, Site Investigator), Toshio Kawamata (Kobe University, Site Investigator), Kazuo Sakai (Kobe University, Site Investigator), Haruhiko Oda (Kobe University, Site Investigator), Takashi Sakurai (Kobe University, Site Investigator), Taichi Akisaki (Kobe University, Site Investigator), Mizuho Adachi (Kobe University, Site Investigator), Masako Kuranaga (Kobe University, Site Investigator), Sachi Takegawa (Kobe University, Site Investigator), Yoshihiko Tahara (Kobe University, Site Investigator), Seishi Terada (Okayama University, Site Investigator), Takeshi Ishihara (Kawasaki Hospital, Site Investigator), Hajime Honda (Okayama University, Site Investigator), Osamu Yokota (Okayama University, Site Investigator), Yuki Kishimoto (Okayama University, Site Investigator), Naoya Takeda (Okayama University, Site Investigator), Nao Imai (Okayama University, Site Investigator), Mayumi Yabe (Okayama University, Site Investigator), Kentaro Ida (Okayama Diagnostic Imaging Center, Site Investigator), Daigo Anami (Okayama Diagnostic Imaging Center, Site Investigator), Seiji Inoue (Okayama Diagnostic Imaging Center, Site Investigator), Toshi Matsushita (Okayama Diagnostic Imaging Center, Site Investigator), Reiko Wada (Okayama University, Site Investigator), Shinsuke Hiramatsu (Okayama Diagnostic Imaging Center, Site Investigator), Hiromi Tonbara (Okayama University, Site Investigator), Reiko Yamamoto (Okayama University, Site Investigator), Kenji Nakashima (Tottori University, Site Investigator), Kenji Wada-Isoe (Tottori University, Site Investigator), Saori Yamasaki (Tottori University, Site Investigator), Eijiro Yamashita (Tottori University, Site Investigator), Yu Nakamura (Kagawa University, Site Investigator), Ichiro Ishikawa (Kagawa University, Site Investigator), Sonoko Danjo (Kagawa University, Site Investigator), Tomomi Shinohara (Kagawa University, Site Investigator), Miyuki Ueno (Kagawa University, Site Investigator), Yuka Kashimoto (Kagawa University, Site Investigator), Yoshihiro Nishiyama (Kagawa University, Site Investigator), Yuka Yamamoto (Kagawa University, Site Investigator), Narihide Kimura (Kagawa University, Site Investigator), Kazuo Ogawa (Kagawa University, Site Investigator), Yasuhiro Sasakawa (Kagawa University, Site Investigator), Takashi Ishimori (Kagawa University, Site Investigator), Yukito Maeda (Kagawa University, Site Investigator), Tatsuo Yamada (Fukuoka University, Site Investigator), Shinji Ouma (Fukuoka University, Site Investigator), Aika Fukuhara -Kaneumi (Fukuoka University, Site Investigator), Nami Sakamoto (Neues Corporation, Site Investigator), Rie Nagao (Neues Corporation, Site Investigator), Kengo Yoshimitsu (Fukuoka University, Site Investigator), Yasuo Kuwabara (Fukuoka University, Site Investigator), Ryuji Nakamuta (Fukuoka University, Site Investigator), Minoru Tanaka (Fukuoka University, Site Investigator), Manabu Ikeda (Kumamoto University, Site Investigator), Mamoru Hashimoto (Kumamoto University, Site Investigator), Keiichirou Kaneda (Kumamoto University, Site Investigator), Yusuke Yatabe (Kumamoto University, Site Investigator), Kazuki Honda (Kumamoto University, Site Investigator), Naoko Ichimi (Kumamoto University, Site Investigator), Fumi Akatuka (Center of support for child development, Site Investigator), Mariko Morinaga (Kumamoto University, Site Investigator), Miyako Noda (Kumamoto University, Site Investigator), Mika Kitajima (Kumamoto University, Site Investigator), Toshinori Hirai (Kumamoto University, Site Investigator), Shinya Shiraishi (Kumamoto University, Site Investigator), Naoji Amano (Shinshu University, Site Investigator), Shinsuke Washizuka (Shinshu University, Site Investigator), Toru Takahashi (Shinshu University, Site Investigator), Shin Inuzuka (Shinshu University, Site Investigator), Tetsuya Hagiwara (Shinshu University, Site Investigator), Nobuhiro Sugiyama (Shinshu University, Site Investigator), Yatsuka Okada (Shinshu University, Site Investigator), Tomomi Ogihara (Shinshu University, Site Investigator), Takehiko Yasaki (Shinshu University, Site Investigator), Minori Kitayama (Shinshu University, Site Investigator), Tomonori Owa (Shinshu University, Site Investigator), Akiko Ryokawa (Shinshu University, Site Investigator), Rie Takeuchi (Shinshu University, Site Investigator), Satoe Goto (Shinshu University, Site Investigator), Keiko Yamauchi (Shinshu University, Site Investigator), Mie Ito (Shinshu University, Site Investigator), Tomoki Kaneko (Shinshu University, Site Investigator), Hitoshi Ueda (Shinshu University, Site Investigator), Shuichi Ikeda (Shinshu University, Site Investigator), Masaki Takao (Mihara Memorial Hospital, Site Investigator), Ban Mihara (Mihara Memorial Hospital, Site Investigator), Hirofumi Kubo (Mihara Memorial Hospital, Site Investigator), Akiko Takano (Mihara Memorial Hospital, Site Investigator), Gou Yasui (Mihara Memorial Hospital, Site Investigator), Masami Akuzawa (Mihara Memorial Hospital, Site Investigator), Kaori Yamaguchi (Mihara Memorial Hospital, Site Investigator), Toshinari Odawara (Yokohama City University, Site Investigator), Megumi Shimamura (Yokohama City University, Site Investigator), Mikiko Sugiyama (Yokohama City University, Site Investigator), Atsushi Watanabe (Yokohama City University, Site Investigator), Naomi Oota (Yokohama City University, Site Investigator), Shigeo Takebayashi (Yokohama City University, Site Investigator), Yoshigazu Hayakawa (Yokohama City University, Site Investigator), Mitsuhiro Idegawa (Yokohama City University, Site Investigator), Noriko Toya (Yokohama City University, Site Investigator)
