# Supplementary material for: Visualizing modules of coordinated structural brain atrophy during the course of conversion to Alzheimer's disease by applying methodology from gene co-expression analysis
Source: Neuroimage Clin. 2019 Jul 25;24:101957. doi: 10.1016/j.nicl.2019.101957 (PMC6700430; doi:10.1016/j.nicl.2019.101957)
Supplement: Supplementary file 2 — Supplementary material 2. Supplemental Table 1-3 [file mmc2.docx]

Supplemental Table 1. Correlation coefficient between regional/structural atrophy and incidence of conversion/ADAS-cog13 progression speed

|  | Baseline dataset (n=204) | | Subtracted dataset (n=100) | |
| --- | --- | --- | --- | --- |
|  | Conversion | ADAS progression | Conversion | ADAS progression |
| entorhinal.x | -0.224 | -0.295 | n.s. | -0.338 |
| entorhinal.y | -0.263 | -0.309 | n.s. | -0.344 |
| fusiform.x | n.s. | -0.275 | n.s. | n.s. |
| inferiortemporal.x | n.s. | -0.242 | n.s. | n.s. |
| middletemporal.x | n.s. | -0.262 | n.s. | n.s. |
| temporalpole.x | n.s. | -0.224 | n.s. | n.s. |
| parahippocampal.y | n.s. | n.s. | -0.37 | -0.413 |
| Right.Hippocampus | -0.313 | -0.247 | n.s. | n.s. |
| Left.Hippocampus | -0.34 | -0.242 | n.s. | n.s. |
| Right.Amygdala | -0.231 | n.s. | n.s. | n.s. |
| Left.Amygdala | -0.283 | n.s. | n.s. | n.s. |
| wm.lh.isthmuscingulate | n.s. | n.s. | n.s. | -0.331 |
| Left.Lateral.Ventricle | n.s. | n.s. | 0.379 | n.s. |
| 3rd.Ventricle | n.s. | n.s. | 0.321 | n.s. |
| Right.Lateral.Ventricle | n.s. | n.s. | 0.380 | 0.335 |

Correlation coefficient results of regions/structures that are significantly correlated with (FDR < 0.05: Spearman’s rank correlation, corrected using the BH method) either the incidence of conversion or ADAS progression speed, are only included in this table. “n.s.” means non-significant.

Supplemental Table 2. Variable importance table of top 20 regions/structures in the baseline dataset (n=204)

|  | Conversion | ADAS-cog13 progression speed | Averaged |
| --- | --- | --- | --- |
| Left.Hippocampus | 100.00 | 30.13 | 65.06 |
| fusiform.x | 39.52 | 84.17 | 61.85 |
| Left.Amygdala | 76.76 | 40.43 | 58.59 |
| inferiortemporal.x | 45.02 | 67.23 | 56.13 |
| entorhinal.y | 55.69 | 53.24 | 54.46 |
| wm.lh.parahippocampal | 7.15 | 100.00 | 53.57 |
| Right.Hippocampus | 81.09 | 24.27 | 52.68 |
| entorhinal.x | 49.99 | 53.93 | 51.96 |
| Right.Amygdala | 71.37 | 32.28 | 51.82 |
| middletemporal.x | 31.25 | 69.26 | 50.25 |
| wm.rh.transversetemporal | 54.23 | 42.16 | 48.20 |
| temporalpole.x | 20.91 | 66.42 | 43.67 |
| wm.rh.paracentral | 56.34 | 27.50 | 41.92 |
| Right.Accumbens.area | 46.09 | 30.86 | 38.47 |
| wm.rh.precuneus | 58.39 | 17.84 | 38.12 |
| wm.lh.lingual | 19.26 | 56.52 | 37.89 |
| Right.UnsegmentedWhiteMatter | 32.96 | 42.24 | 37.60 |
| Left.Accumbens.area | 32.97 | 41.55 | 37.26 |
| fusiform.y | 25.36 | 48.52 | 36.94 |
| wm.rh.temporalpole | 21.81 | 51.28 | 36.55 |

Variable importance of each region/structure within the baseline dataset in the regression for MCI conversion (middle column) or ADAS-cog13 progression speed (right column) identified using a support vector machine. Listed in the order of averaged importance values.

Supplemental Table 3. Variable importance table of top 20 regions/structures in the subtracted dataset (n=100)

|  | Conversion | ADAS-cog13 progression speed | Averaged |
| --- | --- | --- | --- |
| parahippocampal.y | 75.77 | 100.00 | 87.88 |
| Left.Lateral.Ventricle | 100.00 | 65.29 | 82.64 |
| Right.Lateral.Ventricle | 94.51 | 64.77 | 79.64 |
| entorhinal.y | 73.61 | 61.63 | 67.62 |
| inferiortemporal.x | 79.32 | 50.61 | 64.96 |
| 3rd.Ventricle | 70.63 | 52.64 | 61.63 |
| superiortemporal.x | 84.42 | 30.16 | 57.29 |
| wm.lh.isthmuscingulate | 46.30 | 68.16 | 57.23 |
| inferiortemporal.y | 82.80 | 31.37 | 57.08 |
| supramarginal.x | 36.19 | 76.48 | 56.34 |
| Left.Amygdala | 75.94 | 34.12 | 55.03 |
| superiortemporal.y | 89.12 | 19.81 | 54.46 |
| insula.x | 62.21 | 45.66 | 53.93 |
| middletemporal.x | 75.10 | 27.10 | 51.10 |
| entorhinal.x | 48.63 | 53.09 | 50.86 |
| inferiorparietal.y | 89.96 | 10.24 | 50.10 |
| fusiform.y | 67.26 | 32.25 | 49.76 |
| temporalpole.x | 58.83 | 38.89 | 48.86 |
| CC_Anterior | 68.01 | 26.44 | 47.23 |
| rostralmiddlefrontal.x | 62.66 | 31.33 | 47.00 |

Variable importance of each region/structure within the baseline dataset in the regression for MCI conversion (middle column) or ADAS-cog13 progression speed (right column) identified using a support vector machine. Listed in the order of averaged importance values.
